# Voluntary control of wearable robotic exoskeletons by patients with paresis via neuromechanical modeling

**DOI:** 10.1186/s12984-019-0559-z

**Published:** 2019-07-17

**Authors:** Guillaume Durandau, Dario Farina, Guillermo Asín-Prieto, Iris Dimbwadyo-Terrer, Sergio Lerma-Lara, Jose L. Pons, Juan C. Moreno, Massimo Sartori

**Affiliations:** 1 Faculty of Engineering Technology, Department of Biomechanical Engineering, University of Twente, Technical Medical Centre, Building: Horsting. Room: W106, P.O. Box: 217, 7500 AE Enschede, The Netherlands; 2 Department of Bioengineering, Imperial College London, London, UK; 3 Neural Rehabilitation Group, Cajal Institute, Spanish National Research Council, Madrid, Spain; 4 Occupational Thinks Research Group, Centro Superior de Estudios Universitarios La Salle, Universidad Autónoma de Madrid, Madrid, Spain

**Keywords:** Electromyography, EMG-driven modeling, Neuromechanical modeling, Neuromuscular injury, Robotic exoskeleton

## Abstract

**Background:**

Research efforts in neurorehabilitation technologies have been directed towards creating robotic exoskeletons to restore motor function in impaired individuals. However, despite advances in mechatronics and bioelectrical signal processing, current robotic exoskeletons have had only modest clinical impact. A major limitation is the inability to enable exoskeleton voluntary control in neurologically impaired individuals. This hinders the possibility of optimally inducing the activity-driven neuroplastic changes that are required for recovery.

**Methods:**

We have developed a patient-specific computational model of the human musculoskeletal system controlled via neural surrogates, i.e., electromyography-derived neural activations to muscles. The electromyography-driven musculoskeletal model was synthesized into a human-machine interface (HMI) that enabled poststroke and incomplete spinal cord injury patients to voluntarily control multiple joints in a multifunctional robotic exoskeleton in real time.

**Results:**

We demonstrated patients’ control accuracy across a wide range of lower-extremity motor tasks. Remarkably, an increased level of exoskeleton assistance always resulted in a reduction in both amplitude and variability in muscle activations as well as in the mechanical moments required to perform a motor task. Since small discrepancies in onset time between human limb movement and that of the parallel exoskeleton would potentially increase human neuromuscular effort, these results demonstrate that the developed HMI precisely synchronizes the device actuation with residual voluntary muscle contraction capacity in neurologically impaired patients.

**Conclusions:**

Continuous voluntary control of robotic exoskeletons (i.e. event-free and task-independent) has never been demonstrated before in populations with paretic and spastic-like muscle activity, such as those investigated in this study. Our proposed methodology may open new avenues for harnessing residual neuromuscular function in neurologically impaired individuals via symbiotic wearable robots.

**Electronic supplementary material:**

The online version of this article (10.1186/s12984-019-0559-z) contains supplementary material, which is available to authorized users.

## Background

The ability to walk directly relates to quality of life. Neurological lesions such as those underlying stroke and spinal cord injury (SCI) often result in severe motor impairments (i.e., paresis, spasticity, abnormal joint couplings) that compromise an individual’s motor capacity and health throughout the life span. For several decades, scientific effort in rehabilitation robotics has been directed towards exoskeletons that can help enhance motor capacity in neurologically impaired individuals. However, despite advances in mechatronics and bioelectrical signal processing, current robotic exoskeletons have had limited performance when tested in healthy individuals [1] and have achieved only modest clinical impact in neurologically impaired patients [2], e.g., stroke [3, 4], SCI patients [5].

Two major challenges are hampering progress. The first is the inability of current systems to enable an individual patient to voluntarily control the robotic device while inducing positive modulation of neuromuscular activity. This prevents wearable robots from facilitating the activity-driven neuroplastic changes that are required for recovery [6, 7]. The second is an incomplete understanding of how lesions in the central nervous system (CNS) impact musculoskeletal system function, which impedes understanding how patients’ motor intentions should be best supported by a robotic device.

Following a brain lesion, secondary adaptation processes occur in the entire musculoskeletal system, i.e., alterations of muscles, ligaments and tendons properties [8]. In stroke survivors, this results in stiffness and disruption of muscle tone [9] followed by abnormal muscle contractile dynamics and consequent changes in locomotion control paradigms [2]. The scarcity of knowledge regarding the adaptation mechanisms taking place in the composite neuromusculoskeletal system has limited our ability to understand what drives impairment and therefore how to restore lost motor capacity via wearable robots. The development of human-machine interfaces (HMIs) that can take into account individual patients’ neuromuscular alterations is fundamental for enhancing the motor function of neurologically impaired patients [7].

HMIs in commercially available robotic exoskeletons for neurorehabilitation (e.g., Rewalk [10], Lokomat [11] and LOPES [12]) largely rely on position and impedance control [13, 14]. In these approaches, the robotic exoskeleton creates joint trajectories or force fields along predefined kinematic profiles previously extracted from healthy populations [15, 16]. However, this does not fully engage the patient, hampering the emergence of positive neuroplasticity [17], with limited rehabilitation outcomes with respect to conventional therapy [4, 5]. Noncommercial robotic exoskeletons use more complex schemes including HMIs inspired by mechanical principles, e.g., predefined moment patterns triggered at specific phases of the gait cycle [18–20]. These approaches were recently applied to poststroke and cerebral palsy individuals [19, 21, 22]. However, such approaches are limited to supporting a cyclic gait under specific patterns and speeds, thus limiting the patient’s self-pacing and voluntary control of the exoskeleton. A generalization of these methods was proposed via human-in-the-loop paradigms where moment patterns for the exoskeleton are optimized online to reduce the metabolic cost of locomotion [1]. Human-in-the-loop optimization, however, operates on large time scales. That is, for the wearable robot to react and adapt to movement variations, the controller needs to process several minutes of metabolic data (i.e., > 20 min), limiting current applications to healthy individuals only. Alternative approaches use sensitivity amplification control algorithms, where exoskeleton sensory information (i.e., interaction forces) is used to generate control commands [23, 24]. However, this paradigm does not provide support until the patient has produced detectable mechanical force or movement, thus critical in severely impaired individuals [25]. As a result, this has not been employed in patients to provide neuromuscular effort reduction [26].

Other HMI schemes rely on bioelectrical signals recorded from muscles or brain areas [27]. These methods could potentially enable exoskeletons to promptly respond and adapt to the patient’s motor intention, a central aspect of neurorehabilitation robotics [2, 7, 25]. Current schemes include neuro-fuzzy approaches [28] or proportional myocontrol methods [29–31] that use electromyograms (EMGs), sometimes in conjunction with foot-ground reaction forces, to generate direct control commands. However, these methods do not account for the nonlinearity between EMG amplitude and muscle mechanical force, the effect of which is especially important in neurorehabilitation [32–34]. As a result, these methods would not always enable optimal computation of exoskeleton assistive moments proportionally to the patient’s force-generating capacity [35, 36]. This ultimately hinders patient-machine synchronization and limits the patient’s ability to control the exoskeleton voluntarily. Moreover, methods based on proportional myocontrol and foot-ground reaction forces [29, 30] are designed for cyclic locomotion where the subject receives support during a specific part of the gait cycle. These methods rely on detection of pre-defined gait events (e.g. foot-ground contact) and are tuned for a specific motor task (e.g. ground-level locomotion) and joint. Overall, this does not enable continuous (event-free and task-independent) control of robotic exoskeletons. Alternative bioelectrical signals such as electroencephalograms [37–40] are currently limited in the context of robotic exoskeletons due to signal high sensitivity to movement artifacts [41].

We have developed an HMI based on EMG-driven musculoskeletal modeling. This approach accounts for the form and function of the human neuromusculoskeletal system in neurologically impaired patients with paresis. We tested it in a wheelchair-bound patient with incomplete SCI and in two chronic hemiparetic poststroke survivors with residual walking capabilities. Although EMG-driven musculoskeletal modeling was previously employed in conjunction with robotic devices [42–44] it was never applied to neurologically impaired individuals to demonstrate neuromuscular activity reduction. To the best of our knowledge, our results demonstrate the first model-based HMI that enables neurologically impaired patients to voluntarily control multiple degrees of freedoms (DOFs) in complex robotic exoskeletons. Importantly, the results demonstrate that increased levels of exoskeleton assistance induced a positive modulation of neuromuscular activity across a large repertoire of motor tasks. This was reflected in a reduction in both the amplitude and the variability of muscle activations as well as in the resulting human joint moments required to perform a motor task. Since small discrepancies in onset time between human limb movement and that of the parallel exoskeleton can significantly increase human muscle effort [1], our results demonstrate that the proposed approach can precisely synchronize device actuation with human muscle contraction, which is especially challenging in pathological populations with paretic and spastic-like muscle activity.

With neurorehabilitation in mind, it is important stressing that the goal of our HMI is not that of reducing the operator’s EMGs per se. The goal is rather that of amplifying the subject’s force-generating capacity to enable the mechanical moments necessary to execute motor tasks that could not otherwise be performed without the support of the exoskeleton. The overarching goal of the experiments presented in this paper was to enable neurologically impaired patients to voluntarily control a multi-DOF robotic exoskeleton while receiving positive physical assistance [15]. In particular, the major objectives of this study were to test whether I) both healthy subjects and neurologically impaired patients could voluntarily control the angular position of the exoskeleton multiple joints accurately and II) whether our proposed framework could modulate the neuromuscular activity of healthy subjects and neurologically impaired patients (i.e., their muscle activations and resulting moments) as a function of different exoskeleton assistance levels with no loss of joint control accuracy.

## Methods

**Fig. 1:**
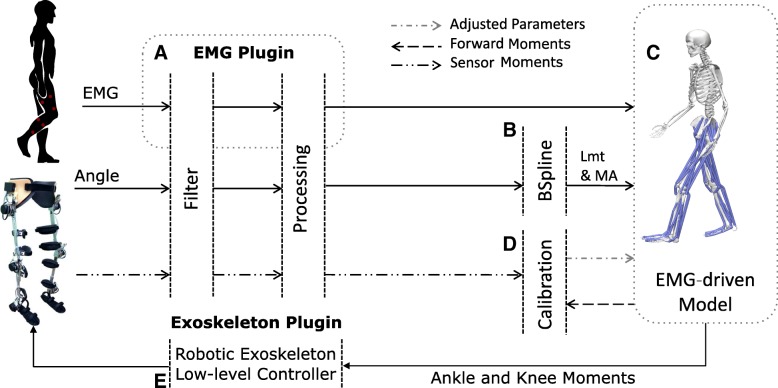
Schematic representation of the real-time modeling framework and its communication with the robotic exoskeleton. The whole framework is operated by a Raspberry Pi 3 single-board computer. The framework consists of five main components: **a** The EMG plugin collects muscle bioelectric signals from wearable active electrodes and transfers them to the EMG-driven model. **b** The B-spline component computes musculotendon length (Lmt) and moment arm (MA) values from joint angles collected via robotic exoskeleton sensors. **c** The EMG-driven model uses input EMG, Lmt and MA data to compute the resulting mechanical forces in 12 lower-extremity musculotendon units (Table 1) and joint moment about the degrees of freedom of knee flexion-extension and ankle plantar-dorsiflexion. **d** The offline calibration procedure identifies internal parameters of the model that vary non-linearly across individuals. These include optimal fiber length and tendon slack length, muscle maximal isometric force, and excitation-to-activation shape factors. **e** The exoskeleton plugin converts EMG-driven model-based joint moment estimates into exoskeleton control commands. Please refer to the Methods section for an in-depth description

We developed a computational patient-specific model of the human lower-extremity musculoskeletal system (Fig. 1). This enabled estimating the mechanical force produced in 12 lower-extremity musculotendon units (MTUs, Table 1) as well as the resulting moments about knee flexion-extension and ankle plantar-dorsiflexion DOFs. Subject-specific models were built individually for four healthy individuals, one incomplete SCI patient and two hemiparetic stroke patients (Table 2).

**Table 1:** EMG-to-MTU mapping

Recorded muscle EMG	Musculotendon unit (MTU)
*Gastrocnemius medialis*	gastrocnemius lateralis and gastrocnemius medialis
*Tibialis anterior*	tibialis anterior
*Soleus*	soleus
*Vastus lateralis*	vastus intermedius, vastus lateralis
*Vastus medialis*	vastus intermedius, vastus medialis
*Semimembranosus*	semimembranosus, semitendinosus
*Biceps femoris*	biceps femoris short head, biceps femoris long head
*Rectus femoris*	rectus femoris

Muscle groups from which experimental electromyography (*EMG*) signals were recorded and the associated musculotendon units (*MTUs*) in the computational modeling framework (Fig. 1c) that were driven by these EMG signals. In this study, the gastrocnemius medialis EMG also drove the gastrocnemius lateralis MTUs. The vastus intermedius EMG activity was calculated as the mean between the vastus lateralis and vastus medialis EMG signals. The long head and short head of the biceps femoris were driven by the same EMG signal. The same applied to the semimembranosus and semitendinosus

**Table 2:** Characteristics of the subjects investigated

Subject	Age (years)	Weight (kg)	Height (cm)	Physical condition	Duration of impairment
Healthy 1	27	75	177	N/A	N/A
Healthy 2	34	67	182	N/A	N/A
Healthy 3	30	73	171	N/A	N/A
Healthy 4	31	82	182	N/A	N/A
Stroke 1	72	50	162	Ischemic Stroke	43 months
Stroke 2	37	85	165	Hemorrhagic Stroke	15 months
SCI	34	75	168	Incomplete SCI	38 months

Descriptions of healthy individuals and recruited stroke and spinal cord injury (SCI) patients

We demonstrated the feasibility of using real-time model-based joint moment estimates for the voluntary control of a robotic exoskeleton throughout a large repertoire of ankle-knee motor tasks. Our proposed framework schematic is depicted in Fig. 1. First, we describe the computational modeling framework structure. Then, we describe the movement data recording and testing protocol.

### Computational modeling framework

#### EMG-driven musculoskeletal modeling framework

We developed an online framework that computes joint moments from EMG signals based on our previous works [45–47]. The framework comprises five main components (Fig. 1). The EMG plugin component (Fig. 1a) provides direct TCP/IP connection with the external EMG system. It records EMGs produced by active electrodes and sends them to the EMG-driven model component.

The *exoskeleton plugin component* (Fig. 1b) records exoskeleton sensory information including human-exoskeleton interaction moments via strain gauges placed on every DOF, as well as the motor moments recorded via motor currents. This plugin assures the transmission of joint moments estimated via the EMG-driven model to the robotic exoskeleton.

The *musculotendon kinematics component* (Fig. 1c) synthesizes MTU paths defined in the subject-specific geometry model (see Experimental procedures section below) into a set of MTU-specific multidimensional cubic B-splines. Each B-spline computes MTU length and moment arms as a function of input joint angles [48]. It enables fast and accurate computation of smooth MTU kinematics, which is central for subsequent moment calculation [48].

The *EMG-driven model component* (Fig. 1c) converts eight input EMG signals into neural activation for 12 MTUs, as presented in Table 1. This is done using a 2nd-order twitch model (Eq. 1) and a nonlinear transfer function (Eq. 2) [35, 47]:

1 $$ {u}_j(t)=\alpha {e}_j\left(t-d\right)-{\beta}_1{u}_j\left(t-1\right)-{\beta}_2{u}_j\left(t-2\right) $$

2 $$ {a}_j(t)=\frac{e^{A{u}_j(t)}-1}{e^A-1} $$

where *u*_*j*_(*t*) is the postprocessed EMG, *e*_*j*_(*t*) is the filtered EMG, *α* is the filtering gain coefficient, *β*_1_ and *β*_2_ are the recursive coefficients, *d* is the electromechanical delay, *a*_*j*_(*t*) is the muscle activation, *A* is the nonlinear shape factor and *j* is the muscle index. MTU-specific neural activation is used in combination with muscle and tendon kinematics to solve for the dynamic equilibrium between fibers and series elastic tendons in the computation of muscle force using a Hill-type muscle model (Eq. 3) [49]:

3 $$ {\displaystyle \begin{array}{l}{F}^{mt}={F}^t={F}^m\mathit{\cos}\left(\phi \left({l}^m\right)\right)\\ {}=\left[a(t)f\left({l}^m\right)f\left({v}^m\right)+{f}_p\left({l}^m\right)\right]{F}^{max}\mathit{\cos}\left(\phi \left({l}^m\right)\right)\end{array}} $$

where *F*^mt^ is the muscle-tendon force, *F*^*t*^ is the tendon force, *F*^*m*^ is the fiber force ,*ϕ*(*l*^*m*^) is the pennation angle, *l*^*m*^ is the fiber length, *v*^*m*^ is the fiber velocity, *f*(*l*^*m*^) is the force due to the fiber force-length relationship, *f*(*v*^*m*^) is the force due to the fiber force-velocity relationship, *f*_*p*_(*l*^*m*^) is the force due to the passive force-length relationship and *a*(*t*) is the muscle activation from Eq. 2. Computed muscle-tendon forces are subsequently transferred onto skeletal joints via moment arms (from the musculotendon kinematics component) to compute resulting moments about two sagittal DOFs. These included knee flexion-extension and ankle plantar-dorsiflexion. Estimated joint moments are used to compute exoskeleton control command as follows (Eq. 4):

4 $$ {\tau}_c(t)={M}_j\left({u}_{m_j}(t),{P}_j(t)\right)\ast G $$

where *τ*_*c*_ is the exoskeleton control command, *M*_*j*_ is the estimated moment for joint *j,* u_{m_j} is the postprocessed EMG of muscle *m* spanning joint *j*, *P*_*j*_ is the angular position of joint *j*, and *G* is the gain that determines the exoskeleton assistance level. The gain G, once chosen, remains constant throughout the experiment. Therefore, for a chosen *G*, there is a fixed mapping between EMG and exoskeleton control commands.

The *model-calibration component* (Fig. 1d) identifies subject-specific model parameters that vary nonlinearly across subjects’ anthropometric features and force-generating capacities. These include the muscle twitch activation/deactivation time constants, EMG-to-activation nonlinearity factor, muscle optimal fiber length, tendon slack length, and muscle maximal isometric force. The initial nominal parameters are repeatedly refined as part of a least-squares optimization procedure so that the mismatch between the EMG-driven model’s predicted joint moments and those measured by the strain gauges of the robotic exoskeleton is minimized [50].

The *low-level exoskeleton controller* (Fig. 1e) transfers joint moment estimates to the main exoskeleton PID controller [15], which distributes moment commands to the motor drive and microcontroller (STMicroelectronics, Switzerland) of each joint.

### Communication framework

The whole real-time modeling framework (Fig. 1) operates on a portable low-power embedded system (Raspberry Pi 3, Raspberry Pi Foundation, UK) with a quad-core processing unit (1.20 GHz) and 1 GB of RAM memory. A custom board was built to digitalize EMG data recorded from active sensors with built-in hardware filtering (13E200, OttoBock, Duderstadt, Germany). The custom board was further connected to a wearable computer board through a Serial Peripheral Interface (SPI) bus to enable bidirectional communication. The robotic exoskeleton was connected to the embedded system running the modeling framework via a controller area network (CAN) protocol and a CAN board (Pican2, SP Pang, UK).

### Experimental procedures

Experimental procedures were divided into two parts, conducted on two consecutive days. The first part established the personalized musculoskeletal model and exoskeleton configuration, i.e., identified subject-specific model parameters and alignment of the human-to-exoskeleton DOF center of rotation. The second part encompassed the exoskeleton voluntary control experiments reported in the Results section.

#### First part – musculoskeletal model and exoskeleton personalization

Motion capture data were recorded (150 Hz) using a seven-camera system (BTS S.p.A., Italy) and a set of 29 retroreflective markers placed on anatomic landmarks on the individual’s lower extremities (bilaterally), pelvis, and trunk [46]. Data were recorded during one static anatomical pose and used in conjunction with the open-source software OpenSim [51] to scale a generic model of the human trunk-pelvis-lower extremity musculoskeletal geometry to match the subject’s anthropometric features. The OpenSim musculoskeletal geometry model had five lower-extremity DOFs (per extremity side), including hip flexion-extension, internal-external rotation, adduction-abduction, knee flexion-extension, and ankle plantar-dorsiflexion. The model included 12 musculotendon units (per lower extremity, i.e., Table 1) and was taken from the literature [52]. During the scaling process, virtual markers were placed on the generic musculoskeletal geometry model based on the position of the experimental markers from the static pose. The musculoskeletal geometry model scaling procedure adjusted the anthropomorphic properties of anatomical segments (i.e., size, mass and inertial properties) as well as MTU insertions and origins and MTU-to-bone wrapping points. These properties were linearly scaled on the basis of the relative distances between the actual subjects’ experimental and corresponding virtual markers [51]. Subsequently, musculotendon parameters were identified, including optimal fiber length and tendon slack length. Because these do not scale proportionally across anthropometric profiles, we employed nonlinear optimization [53]. This was used to iteratively adjust both optimal fiber length and tendon slack length to maintain the consistency of the normalized fiber length-joint angle relationship between an individual and a generic musculoskeletal model across the joint range of motion. This provided initial values for the model-calibration procedure (Fig. 1d) described in the Computational modeling framework section.

Subsequently, subjects wore the robotic exoskeleton for joint alignment. EMG signals were measured (1000 Hz) from eight thigh and shank muscles (Table 1) using dry non-disposable bipolar electrodes (13E200 MyoBock, OttoBock Health Care, GmbH, Germany). Each individual was asked to perform maximal voluntary contractions (MVCs) in isometric conditions with the exoskeleton constraining knee and ankle rotations to predefined arrangements: 45 deg. knee flexion and 0 deg. ankle dorsiflexion. EMG electrodes provided on-board hardware preamplification and filtering to generate output linear envelopes. The resulting envelope peak-processed values were used for EMG normalization. EMG peak values were automatically obtained and saved to a file. The associated joint moment produced during these MVC contractions was not recorded and not used in the subsequent experiments.

After MVCs, subjects performed an additional five cycles of isometric knee flexion-extension followed by five cycles of ankle plantar-dorsiflexion with each joint fixed in an angular position corresponding to the middle of its range of motion (ROM). During these tasks, the exoskeleton built-in strain gauges measured the sagittal knee and ankle joint moments exchanged between the user and the exoskeleton structure. The measured moments were used for the model-calibration step (Computational modeling framework section, Fig. 1d). The paretic patients were instructed to reach their maximal moment contraction, whereas healthy subjects were instructed to exert only a fraction of maximal moment (between ±25 and ± 40 Nm) due to strain gauge sensing limits (maximum range of ±50 Nm). After calibration, the gains for different exoskeleton support levels were determined, including low-gain (LG) and high-gain (HG) support (see Additional file 3: Table S2 for the gain selected for the patients). The LG value was tuned to provide a comfortable, perceptible level of assistance. The HG value was manually tuned to achieve an increase of approximately 50% in exoskeleton moment for the same EMG level with respect to the LG condition. These gains were also empirically and individually adjusted, accounting for each subject’s feedback during the outside the exoskeleton (OUT-type) conditions (see section below).

#### Second part – exoskeleton voluntary control tests

The calibrated subject-specific EMG-driven model was employed in real time to test individuals’ voluntary control of the robotic exoskeleton. First, subjects seated in a medical chair outside the robotic exoskeleton. The exoskeleton was firmly secured next to the subject via a custom-made support, i.e., OUT-type tests. This enabled quantifying the influence of our proposed EMG-driven musculoskeletal model alone on both exoskeleton control accuracy and neuromuscular activity, i.e., without the physical support provided to the user’s leg by the robotic exoskeleton. This was central for assessing whether robotic assistance could be determined purely from patients’ voluntary neuromuscular function. Second, the subjects wore the exoskeleton, i.e., inside the exoskeleton (IN-type) condition tests. This enabled observing the behavior of the composite human-exoskeleton system, i.e., our proposed model-based HMI in conjunction with the physical support provided to the human by the robotic exoskeleton. This was used to verify whether human-exoskeleton synchronization could be achieved to enable neuromuscular effort reduction. Both OUT- and IN-type tests included single-DOF (i.e., the ankle or knee joint individually) and multi-DOF control tasks (i.e., the ankle and knee simultaneously).

**Fig. 2:**
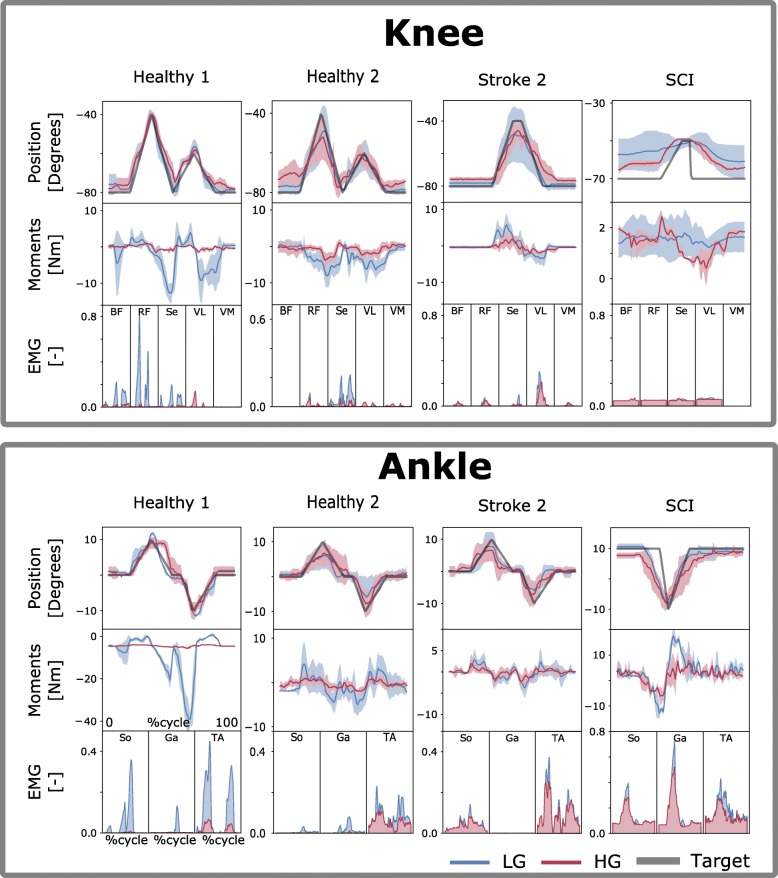
Tracking task performance during single-DOF IN-type tests. Exoskeleton joint angular position, electromyogram (EMG) data and model-based estimates of joint moments are reported during tasks with one degree of freedom (DOF). Data are reported as averages across all tracking trials with standard deviations (shaded area). They are reported for the low-gain (LG) and high-gain (HG) exoskeleton assistance levels and as a function of percent cycle, i.e., where 0 and 100%, respectively, represent the beginning and the end of the tracking trajectory (Target). Target trajectories are personalized to each patient (Table 3) as detailed in the Methods section. The results are relative to tests inside the exoskeleton, i.e., IN-type tests. Data are reported for two representative healthy subjects (Healthy 1–2), two stroke patients (Stroke 1–2) and one incomplete spinal cord injury (SCI) patient. The results are reported both for the individual control of the exoskeleton ankle plantar-dorsiflexion DOF and for that of the exoskeleton knee flexion-extension DOF. EMGs are relative to muscles, including the biceps femoris (BF), rectus femoris (RF), semimembranosus (S), vastus lateralis (VL) and vastus medialis (VM), soleus (So), gastrocnemius medialis (Ga) and tibialis anterior (TA), as shown in Table 1

**Fig. 3:**
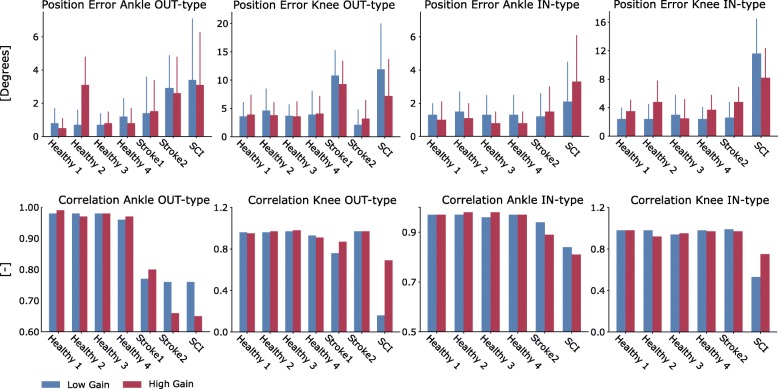
Similarity metrics between reference trajectories and those generated by the controlled exoskeleton. Histograms report root mean square errors (top row, degrees) with standard deviations (bars on top of the histograms) and Pearson correlation coefficients (bottom row) between ensemble average curves obtained for all trials per subject for the specific subject and condition. They are reported for both the low-gain and high-gain assistance levels as well as for individual participants (Table 2) and test types, i.e., the OUT- and IN- types

During the single-DOF tasks, subjects were instructed to perform a series of joint rotations that enabled the exoskeleton joints to be moved to track a monitor-displayed reference trace. Rotations were performed first with the ankle joint and then with the knee joint. For each joint, rotations were performed first with low support gain and then with high support gain. This involved moving the exoskeleton knee or ankle joint to track a joint angle trajectory that spanned a predefined ROM for each joint. ROMs were specifically adjusted for the stroke and SCI patients to avoid joint overextension that would overstretch the muscles, since the patients’ muscles were found to be stiffer than those of healthy individuals (Table 3). Figures 2 and 3 depict the single-DOF trajectories. Each tracking trial was designed to last for 30 s. Each trial was repeated five times.

**Table 3:** Range of motion (in degrees) employed during experiments

Subject	Knee angle (OUT-type)	Ankle angle (OUT-type)	Knee angle (IN-type)	Ankle angle (IN-type)
Healthy	−80 to −40	−10 to 10	−80 to −40	−10 to 10
Stroke 1	−80 to − 40	0 to 15	N/A	N/A
Stroke 2	−80 to −40	−10 to 10	− 80 to − 40	−10 to 10
SCI	−80 to − 40	− 10 to 10	−70 to −50	−10 to 10

Ranges of motion were personalized for each recruited patient to avoid muscle overstretching and testing outside of safe boundaries. Knee flexion and ankle plantar-dorsiflexion are indicated by negative angles

**Fig. 4:**
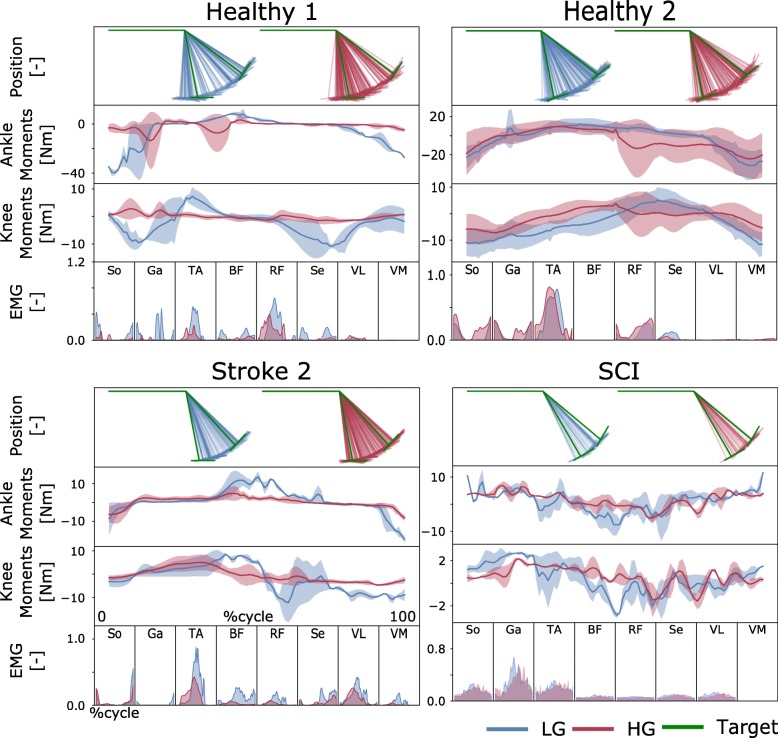
Tracking task performance during multi-DOF IN-type tests. Exoskeleton knee and ankle joint angular positions are reported by means of a stick figure. The green figure represents the target multiple-joint position to be attained. The blue and red stick figures represent the subject’s voluntarily controlled exoskeleton trajectory obtained using low-gain (LG) and high-gain (HG) assistance levels, respectively. The target positions were changed automatically when the user was within ±5 degrees of the target position. Model-based estimates of joint moments are reported about both the knee flexion-extension and ankle plantar-dorsiflexion degrees of freedom (DOFs). Data are reported as averages across all tracking trials with standard deviations (shaded area). They are reported as a function of percent cycle, i.e., where 0 and 100%, respectively, represent the beginning and the end of the tracking trajectory (Target). The results are relative to tests inside of the exoskeleton, i.e., IN-type tests. Data are reported for two representative healthy subjects (Healthy 1–2), one stroke patient (Stroke 2) and one incomplete spinal cord injury (SCI) patient, as shown in Table 2. Recorded electromyograms (EMGs) are relative to muscles including the biceps femoris (BF), rectus femoris (RF), semimembranosus (S), vastus lateralis (VL) and vastus medialis (VM), soleus (So), gastrocnemius medialis (Ga) and tibialis anterior (TA), as presented in Table 1


During the multi-DOF tasks, the reference motions to be tracked involved simultaneous knee flexion-extension and ankle plantar-dorsiflexion. Table 3 reports subject-specific ROMs selected for the participants. Subjects were presented with a graphical user interface displaying real-time information about the kinematic arrangement of the robotic exoskeleton via a stick figure depicted in blue (Fig. 4). A second stick figure, depicted in green, represented the target stick figure to be reached over time (Fig. 4 and Additional file 1: Movie S1). The multi-DOF tests were performed first with low support gain and then with high support gain. Each trial was repeated 5 times for the healthy subjects and up to 5 times for the patients.

**Additional file 1: Movie S1.** Voluntary control of a multijointed robotic exoskeleton by a stroke patient with hemiparesis. A stroke patient with hemiparesis (Stroke 2, Table 2) controls both the ankle and knee joints in a robotic exoskeleton by means of his residual neuromuscular activity. The first part of the movie presents our proposed modeling framework (Fig. 1) working in real time, enabling the patient to exert voluntary control of the exoskeleton knee joint while outside the robotic exoskeleton, i.e., an OUT-type test. The second part of the movie shows the graphical user interface (GUI) we developed to display target trajectories to be tracked by the patient. The third part of the movie presents the patient’s voluntary control of the exoskeleton knee and ankle joints while wearing the exoskeleton, i.e., IN-type tests. (MP4 6312 kb)


### Robotic exoskeleton

All tests were performed using a multi-jointed robotic exoskeleton (H2, Technaid, Spain) equipped with six sagittal non-back-drivable motors (Maxon, Switzerland) with harmonic drive (Harmonic Drive, US), i.e., three motor-drives per leg side. The actuated DOFs were hip flexion-extension (20 deg. flexion, 100 deg. extension), knee flexion-extension (100 deg. flexion, 0 deg. extension), and ankle plantar-dorsiflexion (20 deg. for both plantar flexion and dorsiflexion). The robotic exoskeleton had six strain gauges (i.e., one per joint) for measuring human-exoskeleton joint interacting moments and four foot switches for measuring foot-ground interaction. In this study, we employed a low-level PID controller for each motor that operated in the moment domain. The PID was fine-tuned for moment tracking across a range of mechanical loads [15]. The robotic exoskeleton was powered by a lithium-ion battery with five-hour autonomy.

### Human participants

We recruited four healthy subjects along with three neurologically impaired patients, including one patient with SCI and two chronic hemiparetic stroke patients (Table 2). These patients were selected because representative of the majority of the paretic patient population [54], for whom the effectiveness of rehabilitation robotics with respect to classic rehabilitation has not been demonstrated quantitatively yet [4, 5]. Experiments were conducted on each patient’s most affected side, involving voluntary rotation of two sagittal DOFs in the knee and ankle joints. The dominant leg was used for the experimental tests in healthy individuals. The SCI patient did not participate in the multi-DOF OUT-type test due to time constraints, and stroke patient 1 did not participate in the IN-type test.

### Numerical analysis

We quantified the model real-time performance via mean computation time and standard deviation across all simulation frames from all subjects and tasks. The 95% confidence interval was estimated using Chebyshev’s theorem, i.e., *expected value = mean ± 4.47·std*. This could be applied with no assumptions about the normality of computation time distributions. Similarity metrics between reference and exoskeleton joint kinematic trajectories were assessed via the Pearson product-moment correlation coefficient and the root mean square error for the two considered conditions (IN-type and OUT-type).

Across all tests, data analysis was performed using Python and the NumPy library [55]. In Experiments 1 and 2 (see Results section, Fig. 7, Additional file 3: Figures S3 andS4), we verified whether our framework could induce EMG amplitude reduction while assuring no loss of reference kinematic tracking performance. The histograms (Fig. 7, Additional file 3: Figures S3 and S4) represent the cumulative sum of the mean normalized EMG for each muscle Standard deviation (Eq. 5) was used (Eq. 5) to quantify EMG variability:

5 $$ \sqrt{\frac{\sum \limits_{i=1}^N{\left({x}_i-\overline{x}\right)}^2}{N-1}} $$

where *N* is the number of EMG samples, *x*_*i*_ is the EMG sample for time-frame *i*, and $\overline{x}$ is the mean across all EMG samples. In Experiment 3 (i.e., see Results section, Fig. 6, Additional file 3: Figure S8), overall reduction in EMG variability was visualized via cumulative  standard deviation as well as via normalized cumulative standard deviation. The cumulative EMG variability depicted in Fig. 6 (upper graphs) was computed as follows:

6 $$ \sum \limits_{m=1}^{muscles}{x}_{st{d}_m} $$

where *x_{std_m}* is the standard deviation for muscle *m* computed over all EMG samples and repetitions. The normalized standard deviation for each muscle (Fig. 6, lower graphs) was computed using the following equation:

7 $$ \overline{x_{st{d}_m}}=\frac{x_{st{d}_m}}{x_{mea{n}_m}} $$

where *x_{std_m}* is the same term appearing in Eq. 6 and *x_{mean_m}* is the mean EMG computed across all samples and repetitions for a given muscle.

## Results

### Tracking accuracy of single-DOF trajectories under different assistance levels

**Fig. 5:**
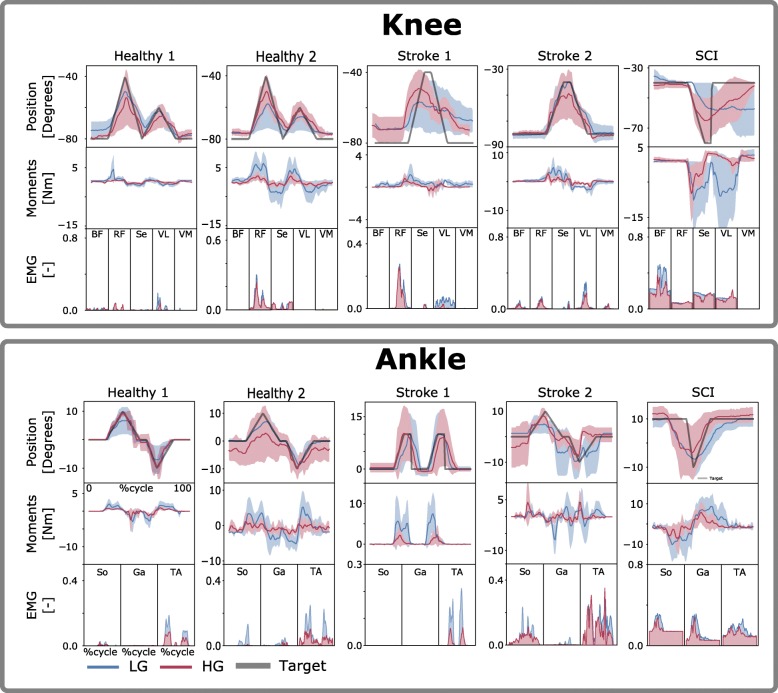
Tracking task performance during single-DOF OUT-type tests. Exoskeleton joint angular position, electromyogram (EMG) data and model-based estimates of joint moments are reported during tasks with one degree of freedom (DOF). Data are reported as averages across all tracking trials with standard deviations (shaded area). They are reported for the low-gain (LG) and high-gain (HG) exoskeleton assistance levels and as a function of percent cycle, i.e., where 0 and 100%, respectively, represent the beginning and the end of the tracking trajectory (Target). Target trajectories are personalized to each patient (Table 3) as detailed in the Methods section. The results are relative to tests outside of the exoskeleton, i.e., OUT-type. Data are reported for two representative healthy subjects (Healthy 1–2), two stroke patients (Stroke 1–2) and one incomplete spinal cord injury (SCI) patient. The results are reported both for the individual control of the exoskeleton ankle plantar-dorsiflexion DOF and for that of the exoskeleton knee flexion-extension DOF. EMGs are relative to muscles including the biceps femoris (BF), rectus femoris (RF), semimembranosus (S), vastus lateralis (VL) and vastus medialis (VM), soleus (So), gastrocnemius medialis (Ga) and tibialis anterior (TA), as shown in Table 1. The EMG for the SCI patient has a large offset due to the high amplification needed

The first test assessed subjects’ ability to control exoskeleton ankle and knee DOFs individually to track monitor-displayed reference trajectories, i.e., Figs. 2 and 5, Additional file 1: Movie S1. This assessed how tracking accuracy varied as a function of increasing robotic exoskeleton assistance levels, i.e., from LG to HG. During single-DOF tasks, the subjects were in full control of the robotic exoskeleton’s knee and ankle DOFs.

Figures 2 and 5, respectively, show results for the OUT- and IN-type tests for both ankle plantar-dorsiflexion and knee flexion-extension. Results are presented for two stroke patients, one SCI patient and two representative healthy subjects. The results from the remaining subjects are reported in Additional file 3: Figures S1 and S2. Figure 3 shows that across all healthy subjects, test types (OUT and IN), DOFs, and gains (LG and HG), the maximal tracking errors were always < 8 degrees with a correlation coefficient always > 0.85. Tracking errors for the patients were on average 5.5 ± 3.1 degrees with correlation coefficients always > 0.6 during IN-type tests. However, during OUT-type tests with LG, tracking errors reached higher values for the SCI patient (i.e., 13 ± 7 degrees) these substantially decreased when employing HG assistance levels (i.e., 6.4 ± 6.1 degrees). Overall, HG assistance offered comparable or reduced tracking capacity with respect to LG assistance but was still within acceptable boundaries (Fig. 3).

Specifically, for the ankle joint during OUT-type tests (Figs. 3 and 5), the mean tracking error and standard deviation (std) across healthy subjects and patients, respectively, measured 1.58 ± 1.64 degrees (LG) and 1.77 ± 1.6 degrees (HG), while the correlation coefficients were 0.88 ± 0.10 (LG) and 0.86 ± 0.14 (HG). During IN-type tests (Figs. 2 and 3), the tracking error measured 1.45 ± 1.35 degrees (LG) and 1.41 ± 1.28 degrees (HG), with correlation coefficients of 0.94 ± 0.04 (LG) and 0.93 ± 0.06 (HG). During the OUT-type tests (Figs. 3 and 5), for the knee joint control, the tracking error measured 5.8 ± 3.98 degrees (LG) and 5.01 ± 3.62 degrees (HG), with correlation coefficients of 0.81 ± 0.27 (LG) and 0.90 ± 0.09 (HG). During IN-type tests (Figs. 2 and 3), the tracking errors measured 4.06 ± 2.55 degrees (LG) and 4.58 ± 2.61 degrees (HG), with correlation coefficients of 0.90 ± 0.16 (LG) and 0.92 ± 0.07 (HG).

### Modulation of neuromuscular activity

The second test quantified the effect of our proposed model-based HMI on the modulation of neuromuscular activity. This was evaluated by examining modulations in normalized EMG and resulting mechanical joint moment amplitudes. Across all experiments, both EMG and resulting moments displayed the largest reduction during the IN-type tests for both healthy participants and patients; see Figs. 4 and 7.

#### Single-DOF experiments

For the ankle joint during OUT-type tests (Fig. 5), the cumulative EMG amplitude (i.e., sum of the mean EMG) decreased for all healthy subjects and patients from LG to HG. The cumulative EMG decreased from 0.04 ± 0.03 to 0.02 ± 0.001 for healthy subject 1, from 0.08 ± 0.04 to 0.04 ± 0.01 for healthy subject 2, from 0.12 ± 0.06 to 0.08 ± 0.04 for healthy subject 3 and from 0.07 ± 0.008 to 0.04 ± 0.004 for healthy subject 4. The cumulative EMG decreased from 0.03 ± 0.02 to 0.01 ± 0.008 for stroke patient 1, from 0.16 ± 0.03 to 0.13 ± 0.08 for stroke patient 2 and from 0.43 ± 0.07 to 0.35 ± 0.04 for the SCI patient.

For the ankle joint during IN-type tests (Fig. 2, Additional file 3: Figure S3), cumulative EMG amplitude decreased from LG to HG, for all healthy subjects and patients, i.e., healthy subject 1 (from 0.16 ± 0.01 to 0.02 ± 0.001), healthy subject 2 (from 0.09 ± 0.04 to 0.06 ± 0.005), healthy subject 3 (from 0.14 ± 0.02 to 0.07 ± 0.06) and healthy subject 4 (i.e., from 0.04 ± 0.03 to 0.01 ± 0.009). For stroke patient 2, we observed a small increase from 0.12 ± 0.06 to 0.13 ± 0.07, whereas the SCI patient showed a small reduction from 0.40 ± 0.03 to 0.39 ± 0.007.

For the knee during OUT-type tests (Fig. 5), we observed decreases or steady values for all healthy subjects and patients. This corresponded to a change in EMG amplitude from 0.04 ± 0.006 to 0.03 ± 0.01 for healthy subject 1, from 0.30 ± 0.03 to 0.12 ± 0.06 for healthy subject 3 and from 0.02 ± 0.02 to 0.04 ± 0.02 for healthy subject 4. For healthy subject 2, we observed an unaltered signal level (i.e., a reduction of 0.001). For users with paresis, we observed reductions from 0.11 ± 0.06 to 0.07 ± 0.02 for stroke patient 1, from 0.11 ± 0.01 to 0.08 ± 0.04 for stroke patient 2 and from 0.65 ± 0.05 to 0.52 ± 0.06 for the SCI patient.

For the knee in the IN-type tests (Fig. 2, Additional file 3: Figure S4), we observed reductions in EMG amplitude for all healthy subjects and patients. Specifically, the amplitude changed from 0.21 ± 0.02 to 0.04 ± 0.003 for healthy subject 1, from 0.07 ± 0.01 to 0.03 ± 0.01 for healthy subject 2, from 0.23 ± 0.04 to 0.12 ± 0.003 for healthy subject 3 and from 0.05 ± 0.01 to 0.02 ± 0.01 for healthy subject 4. Among the patients with paresis, we observed a reduction from 0.09 ± 0.3 to 0.07 ± 0.01 for stroke patient 2 and 0.215 ± 0.01 to 0.209 ± 0.001 for the SCI patient.

Model-based estimates of joint moments were always modulated in response to EMG activity, as shown in Figs. 2 and 5, Additional file 3: Figures S1 and S2. Both knee and ankle joint moments displayed decreases in their mean values from the LG to the HG assistance level. This was observed for both healthy subjects and patients, with the largest reductions observed during the IN-type tests. Additional file 3: Table S1 provides detailed quantitative values.

#### Multi-DOF experiments

This testing condition assessed whether EMG and joint moment amplitude reduction could be observed in tasks relying on larger muscle sets and control of multiple joints. Reference motions to be tracked involved simultaneous knee flexion-extension and ankle plantar-dorsiflexion, as shown in Additional file 2: Movie S2.


**Additional file 2: Movie S2.** Voluntary control of a multijointed robotic exoskeleton by a spinal cord injury patient. A spinal cord injury patient (SCI, Table 2) controls both the ankle and knee joints of a robotic exoskeleton by means of his residual neuromuscular activity. The first part of the movie presents our proposed modeling framework (Fig. 1) working in real time, enabling the patient to exert voluntary control of the exoskeleton knee joint while outside the robotic exoskeleton, i.e., an OUT-type test. The second part of the movie presents the patient’s voluntary control of the exoskeleton knee and ankle joints while wearing the exoskeleton, i.e., IN-type tests. (MP4 6472 kb)


All subjects and patients were able to control the multi-DOF robotic exoskeleton and match the target positions during both OUT-type (Additional file 3: Figures S5-S7) and IN-type tests (Fig. 4). Figure 7 shows that the robotic exoskeleton assistance resulted in a consistent decrease in cumulative EMG amplitude across all subjects. During OUT-type tests (Additional file 3: Figures S5-S7), cumulative EMG amplitude decreased for all healthy subjects and patients between LG and HG. The EMG amplitude decreased for healthy subject 1 (from 0.21 ± 0.02 to 0.12 ± 0.02), subject 2 (from 0.64 ± 0.16 to 0.46 ± 0.27), subject 3 (0.91 ± 0.1 to 0.70 ± 0.23) and subject 4 (from 0.24 ± 0.05 to 0.16 ± 0.02). Among patients, the cumulative EMG amplitude decreased for stroke patient 1 (from 0.20 ± 0.03 to 0.19 ± 0.03) and stroke patient 2 (from 0.35 ± 0.10 to 0.27 ± 0.05). Quantitative data are not available for the SCI patient, who did not perform this test.

During IN-type tests (Figs. 4 and 7, Additional file 3: Figures S5 and S6), increased exoskeleton assistance resulted in EMG reduction for most healthy subjects and all patients, i.e., 0.61 ± 0.003/0.27 ± 0.06 (LG/HG) for healthy subject 1, 0.64 ± 0.19/0.84 ± 0.2 for healthy subject 2, 0.62 ± 0.03/0.25 ± 0.03 for healthy subject 3, 0.21 ± 0.04/0.14 ± 0.03 for healthy subject 4, 0.69 ± 0.14/0.36 ± 0.14 for stroke patient 2 and 0.90 ± 0.18/0.89 ± 0.005 for the SCI patient. Quantitative data are not available for stroke patient 1 who did not perform this test.

Model-based estimates of joint moments were always modulated in response to EMG activity, with knee and ankle joint moments displaying decreases in their mean values from the LG to the HG assistance levels. This was reflected in both healthy subjects and patients, with the largest reductions observed during IN-type tests (Additional file 3: Table S1).

### Variability of neuromuscular activity

**Fig. 6:**
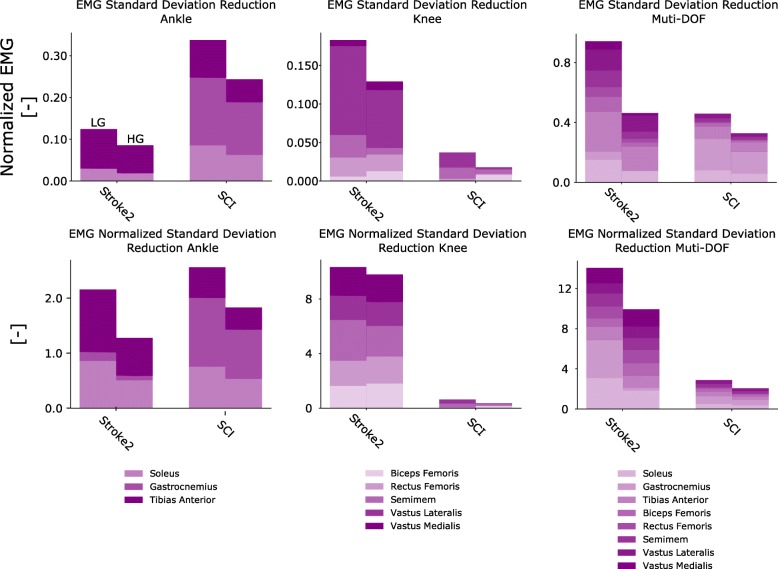
Standard deviation of mean EMG amplitude during single-DOF and multi-DOF IN-type tests. Histograms report the nonnormalized standard deviation (top row) and normalized standard deviation (bottom row, Eq. 7) extracted from electromyogram (EMG) data across all trials performed during single-ankle control tasks, single-knee control tasks and simultaneous ankle-knee control tasks. Histograms are reported relative to low-gain (LG) and high-gain (HG) assistance levels. Data are relative to stroke patient 2 and to the incomplete spinal cord injury patient (SCI), Table 2

The third test assessed the extent of variability in EMG amplitude across exoskeleton assistance levels and tasks, as described by Eq. 6. An index of normalized variability was also computed (Eq. 7) to enable comparison between the LG and HG assistance levels while controlling for mean EMG amplitude. The results showed that increased assistance levels resulted in reduced EMG variability across all patients (Fig. 6, Additional file 3: Figure S8), which may have practical consequences for neurologically impaired patients who are affected by spastic (and thus highly variable) EMG activity.

During OUT-type tests (Additional file 3: Figure S8), EMG variability across all trials (i.e., both single- and multi-DOF) decreased for stroke patient 1 (from 0.48 to 0.38) and stroke patient 2 (from 0.82 to 0.57). After normalization, the variability decreased for stroke patient 2 (from 22.8 to 22.2) and increased for stroke patient 1 (15.2 to 17.2).

During the IN-type tests (Fig. 6), the EMG variability across all trials (i.e., both single- and multi-DOF) decreased for stroke patient 2 (1.24 to 0.67) and for the SCI patient (from 0.83 to 0.58), as shown in the top row histograms in Fig. 6. Similarly, after normalization, the variability decreased for stroke patient 2 (from 26 to 21) and for the SCI patient (from 6 to 4.2), as shown in the bottom row of histograms in Fig. 6.

### Computational time

Across all subjects and tests, the proposed framework generated exoskeleton control commands with an average computational time of 7 ± 3.7 ms – specifically, 5.6 ± 3.3 ms for the EMG-driven modeling and 1.3 ± 0.4 ms for the moment arm and tendon-muscle length. In this study, 95% of the control commands produced in a single time frame were generated within 14 ms. This is well below the length of the muscle electromechanical delay, i.e., ~ 30–80 ms [56], as well as the human perceivable delay in motor execution, i.e., ~ 250 ms [57, 58].

## Discussion

We developed and tested a model-based HMI for the voluntary control of a wearable robotic exoskeleton. We validated it in one wheelchair-bound SCI patient, two hemiparetic chronic stroke patients with residual walking capabilities and four additional healthy individuals. To the best of our knowledge, this study provides the first HMI that enables neurologically impaired patients to voluntarily control multiple DOFs in robotic exoskeletons while inducing a positive modulation of neuromuscular activity, i.e., reduction of both neuromuscular amplitude and variability.

Assessing the benefit of robotic devices for enhancing neurologically impaired patients’ movement is challenging. In this study, we focused on quantifying our model-based HMI ability of modulating EMG amplitude and variability as a function of assistive support levels. The focus was to amplify human mechanical function in a clinically viable way. This was realized by enabling generation of mechanical joint moments using reduced EMG activity via active exoskeleton support.

**Fig. 7:**
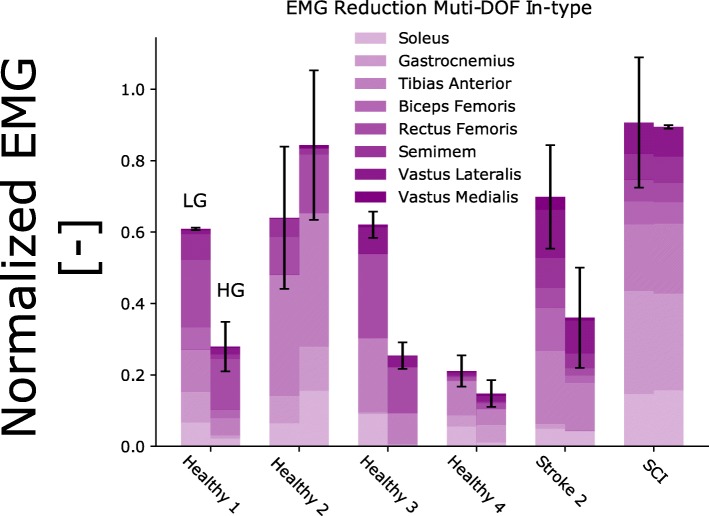
EMG amplitude modulation between low and high levels of exoskeleton assistance during multi-DOF IN-type experiments. The results are reported for tracking tasks with multiple degrees of freedom (DOFs), involving simultaneous knee and ankle joint movement. Subjects performed the experiments while wearing the robotic exoskeleton, i.e., IN-type tests. For each healthy subject (Healthy 1–4) as well as for stroke patient 2 (Stroke) and the spinal cord injury (SCI) patient (Table 2), the vertical bars report the mean normalized EMG amplitude stacked vertically for each muscle along with the standard deviation (see black vertical lines)

We established a numerical model of the human musculoskeletal system that could be scaled and calibrated to match an individual’s anatomy. In this context, experimentally recorded EMG signals represented a surrogate of the neural drive to muscles. That is, EMG linear envelope amplitude and shape features reflected the patient’s disrupted motor control. Moreover, the proposed patient-specific muscle model allowed capturing the patient’s impaired muscular force-generating capacity. Unlike state-of-the-art HMIs, our proposed approach allowed a robotic exoskeleton to be controlled proportionally to an individual patient’s residual muscle force-generating capacity, as shown in Figs. 2, 3, 6 and 7. Furthermore, our method provided an enhanced level of patient specificity with respect to state-of-the-art exoskeleton HMIs.

Results showed that all subjects were able to voluntarily control the exoskeleton accurately over a range of motor tasks involving rotations about single DOFs as well as multiple DOFs concurrently, i.e., Figs. 2, 3, 4 and 7. Subjects could control the exoskeleton to accurately track reference trajectories even when they were not directly wearing the exoskeleton, i.e., during OUT-type tests. This was hypothesized to be a challenging condition due to reduced perception of exoskeleton movement. Overall, results highlighted that patients were always in control of the exoskeleton, thus not being passively driven.

Results showed that an increasing level of assistance induced a decrease in the net cumulative EMG amplitude and resulting mechanical moments required to perform a motor task (Figs. 2, 3, 4, 5 and 7). Reduction was observed across all measured muscles for all subjects both during single-DOF and multi-DOF tasks. However, in a few cases, HG assistance induced higher EMG activity than LG assistance, as shown in Fig. 7, subject 2. Systematic analyses are planned as part of future work to identify direct causes. Importantly, the increased level of assistance did not degrade the user’s accuracy in the reference position tracking tasks (Fig. 3).

The exoskeleton generated a support moment that was equal to a fraction of the user’s net joint moment (calculated by the EMG-driven model). For higher gains the exoskeleton support moment reflected a larger fraction of the user’s net joint moment. Importantly, across support gains, the reference mechanical task to be performed by the patient and exoskeleton system was always the same. That is, the underlying total net moment to be generated by the patient and exoskeleton system was similar across support gains. Therefore, for increasing support gains, the proportion of human-generated moment decreased while the proportion of exoskeleton-generated moment increased. This was directly reflected in our results (Figs. 2, 4 and 5).

Across all subjects, the net EMG amplitude reduction was consistently associated with a concurrent reduction in EMG variability. Motor tasks performed with high assistance levels corresponded to more repeatable EMG patterns than tasks performed with low assistance levels (Fig. 6). This could be achieved only if the exoskeleton joint actuation was precisely synchronized with the patient’s muscle contraction. Lower levels of human-exoskeleton synchronization would lead to the exoskeleton counteracting the patient’s movements or providing suboptimal assistive moment, thereby inducing an increase in EMG magnitude and variability. Personalized models played an important role in achieving these results. Additional file 3: Figure S9 shows how noncalibrated models display large discrepancies with respect to reference moments, which would hamper the controllability of exoskeletons. Patients with neurological lesions naturally present greater movement variability than healthy individuals [59], especially because of involuntary (spastic-like) muscle activity. In this context, spasticity or hyperactive stretch reflexes would be directly captured via EMGs. Because our proposed musculoskeletal model was driven by EMGs, this enabled capturing muscle force controlled by abnormal spinal neuron activity. Our results showed that our proposed model-based amplification of patient’s neuromuscular function enabled tracking smooth join position trajectories despite the underlying patients’ EMGs may underlie spastic activity. This may be due to the fact that the presence of high-energy spikes in the EMG (i.e. due to spastic-like EMGs) may be attenuated by the inherent visco-elasticity of the Hill-type muscle model as well as muscle-tendon small moment arms. This has the benefit of generating smooth muscle force-dependent joint moment profiles even in the presence of spikes in the input EMGs. Future work will systematically assess the ability of musculoskeletal models in attenuating EMG abnormal spiking activity and also assess its robustness with respect to abnormal activation, such as spasticity, in a rehabilitation scenario. Furthermore, future studies will assess the possibility of using high-density EMG recording to decompose the signal at the level of constituent motor unit discharges. This would enable separating physiological motor units from those displaying abnormal behavior (i.e. spastic-like). For each muscle, filtered physiological motor units only could be used to compute exoskeleton assistive moments [35].

The ability to enforce EMG pattern repeatability (i.e., reduction of EMG variability) via wearable robotic technologies is central for retraining coordinated neuromuscular control and inducing positive neuroplasticity, i.e., by preventing involuntary uncoordinated muscle activations [7]. The ability of the proposed HMI to modulate neuromuscular activity in a controlled way may be beneficial in the future for high-intensity sensorimotor training, i.e., enabling patients to perform intensive motor tasks progressively across longer periods of time (EMG reduction). Intensive training has been shown to improve both muscle strength and overall motor control in stroke patients [60].

Our HMI always computed exoskeleton control commands within the muscle electrophysiological delay, i.e., < 15 ms. This computational speed was achieved using a low-power, small-sized, and fully wearable processing unit, i.e., Raspberry Pi 3 (Raspberry Pi Foundation, UK). This enabled the subject’s movement to be predicted shortly before the actual movement took place, which is important for synchronizing the exoskeleton response to the user’s neuromuscular function. Moreover, this was crucial especially for supporting neurologically impaired patients who had severely reduced motor abilities but still had detectable EMG activity. This is an important advantage with respect to HMIs that actuate the wearable robot solely on the basis of the detection of externally measurable forces (i.e., external joint moments or limb-orthosis interaction force); such systems cannot provide support unless the patient is able to produce detectable muscle force or movement [23]. In the context of our experiment, this would have severely challenged both voluntary exoskeleton control and EMG reduction in the SCI and stroke patients, whose muscle strength and EMG amplitude were substantially compromised.

This study involved voluntary control of robotic knee and ankle rotations from a seated position. These exercises were selected for two reasons. First, they provided a controlled environment for testing our proposed methods for the first time in neurologically impaired individuals. Second, they mimicked physiotherapy tasks employed during early-stage rehabilitation. Functional impairment post-stroke underlies loss of selective joint control and muscle weakness. From a clinical perspective, both issues must be addressed via safe, comfortable and feasible positions for the patient [61, 62], something that could be provided by our proposed framework. Future work will pair wearable exoskeletons with our model-based human-machine interface to track and support the patient across all recovery stages: from sitting to walking in the hospital to finally walking outside the hospital [63]. An advantage of our framework over conventional inverse dynamics is that, once calibrated, it does not need ground reaction forces, i.e., it operates as a function of EMG and joint position, which are measurable via wearable sensors. This is central for wearable robotics applications.

To the best of our knowledge, there is currently no robotic exoskeleton on the market, either in the rehabilitation domain (i.e., Lokomat) or in the assistive domains (i.e., Rewalk, HAL), that operates as a function of a patient’s residual muscle force-generating capacity. This may underpin a central element hampering the ability of current robotic exoskeletons to impact neurorehabilitation. Additional file 2: Movie S2 shows the possibility for the SCI patient to control multiple DOFs despite minimal residual motor capacity, which is key for promoting recovery even in severely affected individuals. This study was not intended to quantify direct rehabilitation outcome, as this requires systematic analysis on a larger patient population, which is subject of future work.

Future work will extend our proposed methodology towards more functional tasks, such as ground- and inclined-level walking, as well as stair ascending and descending. We will investigate the effect of the exoskeleton on the patient with and without the device, as well as the effect of ground reaction force on the exoskeleton combined with our framework. A limitation of this study is that we did not test how noncalibrated models would affect exoskeleton control. Longitudinal tests with different types of models (i.e., calibrated and noncalibrated) will be performed as part of our future work. The overall quality of the model optimized parameters (maximal isometric muscle force, optimal fiber length and tendon slack length) should also be validated against in-vivo experimental values in future work. However, it was first necessary to assess whether neurologically impaired patients could achieve voluntary control of robotic knee and ankle rotation with the current framework.

## Conclusion

This study established a new patient-specific model-based HMI that could aid clinicians and physiotherapists in the assessment of patients’ motor capacity and progress over time. It could enable exoskeletons to operate symbiotically to the human body by dynamically adapting to the patient’s motor capacity across different stages of recovery. This will open new avenues for establishing personalized neurorehabilitation technologies where wearable robots physically interact with the patient to maximize the recovery of compromised neuromuscular targets.

## Additional files

Additional file 3: **Figure S1.** Tracking task performance during single-DOF tests for healthy subject 3. **Figure S2.** Tracking task performance during single-DOF tests for healthy subject 4. **Figure S3.** EMG amplitude modulation between exoskeleton low- and high-assistance levels during single ankle plantar-dorsi flexion, IN-type experiments. **Figure S4.** EMG amplitude modulation between exoskeleton low- and high-assistance levels during single knee flexion-extension, IN-type experiments. **Figure S5** Tracking task performance during multi-DOF, OUT- and IN-type tests for healthy subject 3. **Figure S6** Tracking task performance during multi-DOF, OUT- and IN-type tests for healthy subject 4. **Figure S7** Tracking task performance during multi-DOF, OUT-type tests. **Figure S8** Standard deviation of the mean EMG amplitude during single-DOF and multi-DOF, OUT-type tests. **Figure S9** Predicted moment for the Ankle plantar-dorsiflexion and the Knee flexion-extension using an uncalibrated model. **Table S1** Joint moment modulation across assistance levels. **Table S2** Gain used for the assistance for the patients. (PDF 1320 kb)

## Data Availability

Data and code are available upon request.

## Abbreviations

CAN: Controller area network
CNS: Central nervous system
DOF: Degree of freedom
EMG: Electromyogram
HG: High gain
HMI: Human-machine interface
IN: Inside the exoskeleton
LG: Low gain
MTU: Musculotendon unit
MVC: Maximal voluntary contraction
OUT: Outside the exoskeleton
PID: Proportional integral derivative
SCI: Spinal cord injury
std: Standard deviation
TCP/IP: Transmission control protocol/Internet protocol

## References

[1] Zhang J, Fiers P, Witte KA, Jackson RW, Poggensee KL, Atkeson CG (2017). Human-in-the-loop optimization of exoskeleton assistance during walking. Science (80- ).

[2] Reinkensmeyer D, Dietz V (2016). Neurorehabilitation technology, second edition.

[3] Hornby TG, Campbell DD, Kahn JH, Demott T, Moore JL, Roth HR (2008). Enhanced gait-related improvements after therapist- versus robotic-assisted locomotor training in subjects with chronic stroke: a randomized controlled study. Stroke.

[4] Hidler J, Nichols D, Pelliccio M, Brady K, Campbell DD, Kahn JH (2009). Multicenter randomized clinical trial evaluating the effectiveness of the Lokomat in subacute stroke. Neurorehabil Neural Repair.

[5] Swinnen E, Duerinck S, Baeyens J, Meeusen R, Kerckhofs E (2010). Effectiveness of robot-assisted gait training in persons with spinal cord injury: a systematic review. J Rehabil Med.

[6] Dimyan Michael A., Cohen Leonardo G. (2011). Neuroplasticity in the context of motor rehabilitation after stroke. Nature Reviews Neurology.

[7] Cramer SC, Sur M, Dobkin BH, O’Brien C, Sanger TD, Trojanowski JQ (2011). Harnessing neuroplasticity for clinical applications. Brain.

[8] Dietz V (2002). Proprioception and locomotor disorders. Nat Rev Neurosci.

[9] Dietz V, Sinkjaer T (2007). Spastic movement disorder: impaired reflex function and altered muscle mechanics. Lancet Neurol.

[10] Esquenazi A, Talaty M, Packel A, Saulino M (2012). The ReWalk powered exoskeleton to restore ambulatory function to individuals with thoracic-level motor-complete spinal cord injury. Am J Phys Med Rehabil.

[11] Jezernik S, Colombo G, Keller T, Frueh H, Morari M (2003). Robotic orthosis lokomat: a rehabilitation and research tool. Neuromodulation.

[12] Veneman JF, Kruidhof R, Hekman EEG, Ekkelenkamp R, Van Asseldonk EHF, Van Der Kooij H (2007). Design and evaluation of the LOPES exoskeleton robot for interactive gait rehabilitation. IEEE Trans Neural Syst Rehabil Eng.

[13] Westlake KP, Patten C (2009). Pilot study of Lokomat versus manual-assisted treadmill training for locomotor recovery post-stroke. J Neuroeng Rehabil.

[14] Yan T, Cempini M, Oddo CM, Vitiello N (2015). Review of assistive strategies in powered lower-limb orthoses and exoskeletons. Rob Auton Syst.

[15] Bortole M, Venkatakrishnan A, Zhu F, Moreno JC, Francisco GE, Pons JL (2015). The H2 robotic exoskeleton for gait rehabilitation after stroke: early findings from a clinical study. J Neuroeng Rehabil.

[16] Huo Weiguang, Mohammed Samer, Moreno Juan C., Amirat Yacine (2016). Lower Limb Wearable Robots for Assistance and Rehabilitation: A State of the Art. IEEE Systems Journal.

[17] Attneave F, M B, Hebb DO (1950). The organization of behavior; a neuropsychological theory. Am J Psychol.

[18] Panizzolo FA, Galiana I, Asbeck AT, Siviy C, Schmidt K, Holt KG (2016). A biologically-inspired multi-joint soft exosuit that can reduce the energy cost of loaded walking. J Neuroeng Rehabil.

[19] Lerner Zachary F., Damiano Diane L., Bulea Thomas C. (2017). A lower-extremity exoskeleton improves knee extension in children with crouch gait from cerebral palsy. Science Translational Medicine.

[20] Cajigas I, Koenig A, Severini G, Smith M, Bonato P (2017). Robot-induced perturbations of human walking reveal a selective generation of motor adaptation. Sci Robot.

[21] Awad Louis N., Bae Jaehyun, O’Donnell Kathleen, De Rossi Stefano M. M., Hendron Kathryn, Sloot Lizeth H., Kudzia Pawel, Allen Stephen, Holt Kenneth G., Ellis Terry D., Walsh Conor J. (2017). A soft robotic exosuit improves walking in patients after stroke. Science Translational Medicine.

[22] Kang J, Martelli D, Vashista V, Martinez-Hernandez I, Kim H, Agrawal SK (2017). Robot-driven downward pelvic pull to improve crouch gait in children with cerebral palsy. Sci Robot.

[23] Kazerooni H, Racine J-L, Steger R (2005). On the control of the Berkeley lower extremity exoskeleton (BLEEX). Proc 2005 IEEE Int Conf Robot Autom..

[24] Huang R, Cheng H, Chen QM, Tran HT, Lin XC. Interactive learning for sensitivity factors of a human-powered augmentation lower exoskeleton. In: 2015 IEEE/RSJ International Conference on Intelligent Robots and Systems (IROS). Germany: IEEE; 2015. p. 6409–15.

[25] Sartori M, Llyod DG, Farina D (2016). Neural data-driven musculoskeletal modeling for personalized neurorehabilitation technologies. IEEE Trans Biomed Eng.

[26] Yang Z, Gu W, Zhang J, Gui L (2017). Sensitivity amplification control of exoskeleton suit. Force control theory and method of human load carrying exoskeleton suit.

[27] Kawamoto H, Sankai Y (2005). Power assist method based on phase sequence and muscle force condition for HAL. Adv Robot.

[28] Kiguchi K, Tanaka T, Fukuda T (2004). Neuro-fuzzy control of a robotic exoskeleton with EMG signals. Informatica.

[29] Cain SM, Gordon KE, Ferris DP (2007). Locomotor adaptation to a powered ankle-foot orthosis depends on control method. J Neuroeng Rehabil.

[30] Takahashi KZ, Lewek MD, Sawicki GS (2015). A neuromechanics-based powered ankle exoskeleton to assist walking post-stroke: a feasibility study.

[31] McCain EM, Dick TJM, Giest TN, Nuckols RW, Lewek MD, Saul KR (2019). Mechanics and energetics of post-stroke walking aided by a powered ankle exoskeleton with speed-adaptive myoelectric control. J Neuroeng Rehabil.

[32] Tang A, Rymer WZ (1981). Abnormal force--EMG relations in paretic limbs of hemiparetic human subjects. J Neurol Neurosurg Psychiatry.

[33] Zhou P, Suresh NL, Rymer WZ (2007). Model based sensitivity analysis of EMG-force relation with respect to motor unit properties: applications to muscle paresis in stroke. Ann Biomed Eng.

[34] Kiguchi K, Imada Y, Liyanage M. EMG-based neuro-fuzzy control of a 4DOF upper-limb power-assist exoskeleton. In: Annual international conference of the IEEE engineering in medicine and biology - proceedings: IEEE; 2007. p. 3040–3. 10.1109/IEMBS.2007.4352969 18002635

[35] Sartori M, Yavuz US, Farina D (2017). In vivo neuromechanics: decoding causal motor neuron behavior with resulting musculoskeletal function. Sci Rep.

[36] Martinez-Valdes Eduardo, Negro Francesco, Falla Deborah, De Nunzio Alessandro Marco, Farina Dario (2018). Surface electromyographic amplitude does not identify differences in neural drive to synergistic muscles. Journal of Applied Physiology.

[37] Jochumsen M, Niazi IK, Mrachacz-Kersting N, Farina D, Dremstrup K (2013). Detection and classification of movement-related cortical potentials associated with task force and speed. J Neural Eng.

[38] Noda T, Sugimoto N, Furukawa J, Sato MA, Hyon SH, Morimoto J (2012). Brain-controlled exoskeleton robot for BMI rehabilitation. IEEE-RAS Int Conf Humanoid Robot.

[39] Kwak N-S, Müller K-R, Lee S-W (2015). A lower limb exoskeleton control system based on steady state visual evoked potentials. J Neural Eng.

[40] Zhu F, Bortole M, Venkatakrishnan A, Hernandez, Francisco G, Pons J (2014). Human-machine system for the H2 lower limb exoskeleton: neural decoding of robot-assisted walking from scalp EEG for stroke rehabilitation. Neuromodulation.

[41] Úbeda A, Azorín JM, Farina D, Sartori M (2018). Estimation of neuromuscular primitives from EEG slow cortical potentials in incomplete spinal cord injury individuals for a new class of brain-machine interfaces. Front Comput Neurosci.

[42] Fleischer C, Hommel G (2008). A human-exoskeleton interface utilizing electromyography. IEEE Trans Robot.

[43] Cavallaro EE, Rosen J, Perry JC, Burns S (2006). Real-time myoprocessors for a neural controlled powered exoskeleton arm. IEEE Trans Biomed Eng.

[44] Sartori M, Durandau G, Došen S, Farina D (2018). Robust simultaneous myoelectric control of multiple degrees of freedom in wrist-hand prostheses by real-time neuromusculoskeletal modeling. J Neural Eng.

[45] Pizzolato C, Lloyd DG, Sartori M, Ceseracciu E, Besier TF, Fregly BJ (2015). CEINMS: a toolbox to investigate the influence of different neural control solutions on the prediction of muscle excitation and joint moments during dynamic motor tasks. J Biomech.

[46] Sartori Massimo, Reggiani Monica, Farina Dario, Lloyd David G. (2012). EMG-Driven Forward-Dynamic Estimation of Muscle Force and Joint Moment about Multiple Degrees of Freedom in the Human Lower Extremity. PLoS ONE.

[47] Durandau G, Farina D, Sartori M. Robust real-time musculoskeletal modeling driven by electromyograms. IEEE Trans Biomed Eng. 2017. 10.1109/TBME.2017.2704085 28504931

[48] Sartori M, Reggiani M, van den Bogert AJ, Lloyd DG (2012). Estimation of musculotendon kinematics in large musculoskeletal models using multidimensional B-splines. J Biomech.

[49] Sartori M, Maculan M, Pizzolato C, Reggiani M, Farina D, Claudio P (2015). Modeling and simulating the neuromuscular mechanisms regulating ankle and knee joint stiffness during human locomotion. J Neurophysiol.

[50] Sartori M, Farina D, Lloyd DG (2014). Hybrid neuromusculoskeletal modeling to best track joint moments using a balance between muscle excitations derived from electromyograms and optimization. J Biomech.

[51] Delp SL, Anderson FC, Arnold AS, Loan P, Habib A, John CT (2007). OpenSim: open-source software to create and analyze dynamic simulations of movement. Biomed Eng IEEE Trans.

[52] Delp SL, Loan JP, Hoy MG, Zajac FE, Topp EL, Rosen JM (1990). An interactive graphics-based model of the lower extremity to study orthopaedic surgical procedures. IEEE Trans Biomed Eng.

[53] Winby CR, Lloyd DG, Besier TF, Kirk TB (2009). Muscle and external load contribution to knee joint contact loads during normal gait. J Biomech.

[54] Adamson J, Beswick A, Ebrahim S (2004). Is stroke the most common cause of disability?. J Stroke Cerebrovasc Dis.

[55] Oliphant TE (2007). Python for scientific computing. Comput Sci Eng.

[56] Nordez A, Gallot T, Catheline S, Guével A, Cornu C, Hug F (2009). Electromechanical delay revisited using very high frame rate ultrasound. J Appl Physiol.

[57] Farina D, Sartori M. Surface electromyography for man-machine interfacing in rehabilitation technologies. In: Farina D, Merletti R, editors. Surface electromyography: physiology, engineering and applications. 2nd ed: IEEE/Wiley; 2016. p. 540–60.

[58] Parker P, Englehart K, Hudgins B (2006). Myoelectric signal processing for control of powered limb prostheses. J Electromyogr Kinesiol.

[59] Chang S-H, Francisco GE, Zhou P, Rymer WZ, Li S (2013). Spasticity, weakness, force variability and sustained spontaneous motor unit discharges of resting spastic-paretic biceps brachii muscles in chronic stroke. Muscle Nerve.

[60] Volpe BT, Lynch D, Rykman-Berland A, Ferraro M, Galgano M, Hogan N (2008). Intensive sensorimotor arm training mediated by therapist or robot improves hemiparesis in patients with chronic stroke. Neurorehabil Neural Repair.

[61] Cohen JW, Ivanova TD, Brouwer B, Miller KJ, Bryant D, Garland SJ (2018). Do performance measures of strength, balance, and mobility predict quality of life and community reintegration after stroke?. Arch Phys Med Rehabil.

[62] Katzan IL, Thompson NR, Uchino K, Lapin B (2018). The most affected health domains after ischemic stroke. Neurology.

[63] Mahendran N, Kuys SS, Brauer SG (2016). Recovery of ambulation activity across the first six months post-stroke. Gait Posture.

