# Supplementary material for: Voluntary control of wearable robotic exoskeletons by patients with paresis via neuromechanical modeling
Source: J Neuroeng Rehabil. 2019 Jul 17;16:91. doi: 10.1186/s12984-019-0559-z (PMC6637518; doi:10.1186/s12984-019-0559-z)
Supplement: Supplementary file 3 — Figure S1. Tracking task performance during single-DOF tests for healthy subject 3. Figure S2. Tracking task performance during single-DOF tests for healthy subject 4. Figure S3. EMG amplitude modulation between exoskeleton low- and high-assistance levels during single ankle plantar-dorsi flexion, IN-type experiments. Figure S4. EMG amplitude modulation between exoskeleton low- and high-assistance levels during single knee flexion-extension, IN-type experiments. Figure S5 Tracking task performance during multi-DOF, OUT- and IN-type tests for healthy subject 3. Figure S6 Tracking task performance during multi-DOF, OUT- and IN-type tests for healthy subject 4. Figure S7 Tracking task performance during multi-DOF, OUT-type tests. Figure S8 Standard deviation of the mean EMG amplitude during single-DOF and multi-DOF, OUT-type tests. Figure S9 Predicted moment for the Ankle plantar-dorsiflexion and the Knee flexion-extension using an uncalibrated model. Table S1 Joint moment modulation across assistance levels. Table S2 Gain used for the assistance for the patients. (PDF 1320 kb) [file 12984_2019_559_MOESM3_ESM.pdf]

Voluntary control of wearable robotic exoskeletons by patients with paresis via neuromechanical modeling

Guillaume Durandau[1], Dario Farina[2], Guillermo Asín-Prieto[3], Iris Dimbwadyo-Terrer[4], Sergio Lerma-Lara[4], Jose L. Pons[3], Juan C. Moreno[3] and Massimo Sartori[1,*]

[1]Department of Biomechanical Engineering, University of Twente, THE NETHERLAND
[2]Department of Bioengineering, Imperial College London, London, UNITED KINGDOM
[3]Neural Rehabilitation Group, Cajal Institute, Spanish National Research Council, SPAIN
[4]Occupational Thinks Research Group, Centro Superior de Estudios Universitarios La Salle, Universidad Autónoma de Madrid, SPAIN

*Address of correspondence:
Massimo Sartori, Ph.D.
University of Twente
Technical Medical Centre
Faculty of Engineering Technology
Department of Biomechanical Engineering
Building: Horsting. Room: W106. P.O. Box: 217
7500 AE Enschede, The Netherlands
Email: m.sartori@utwente.nl

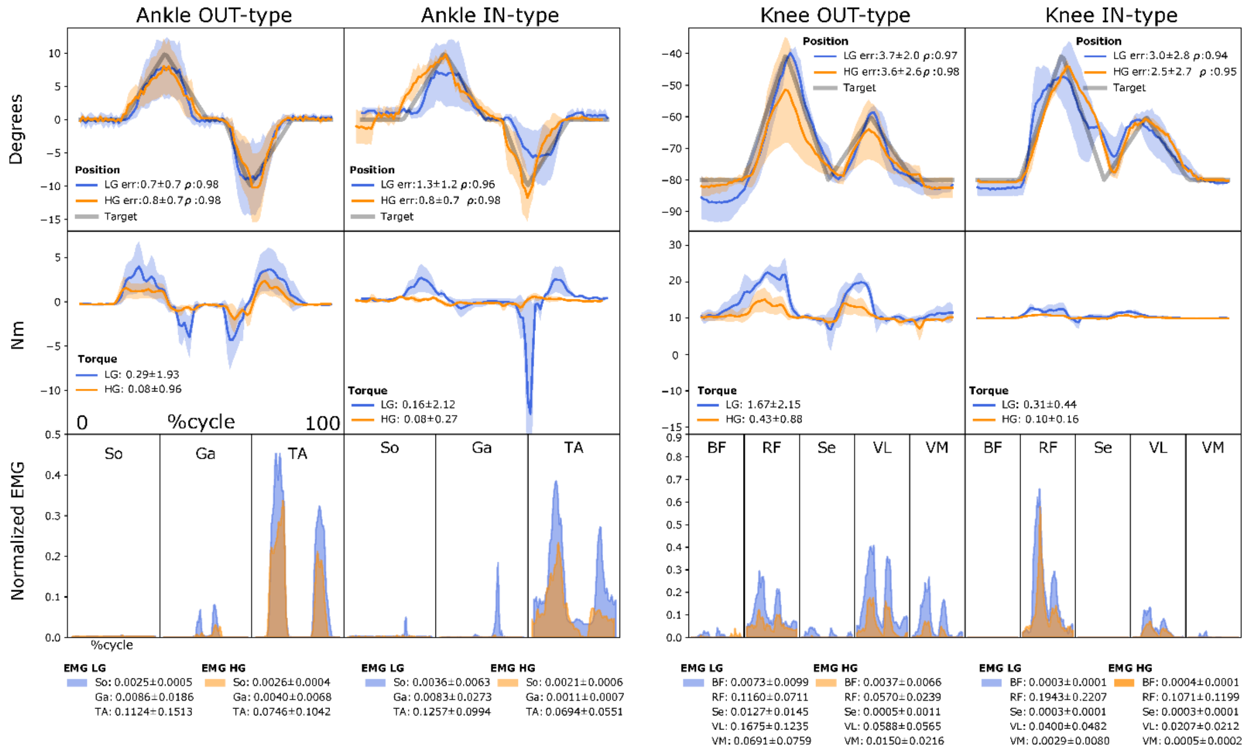

Supplementary figure S1. Tracking task performance during single-DOF tests for healthy subject 3. Exoskeleton joint angular position, electromyography (EMG) data as well as model-based estimates of joint moments (torque) are reported during single degree of freedom (DOF) tasks. Data are reported as averaged across all tracking trials. They are reported for the low-gain (LG) and high-gain (HG) exoskeletons assistance levels and as a function of percent cycle, i.e. where 0% and 100% respectively represents the beginning and the end of the tracking trajectory (Target). Results are relative to tests outside and inside of the exoskeleton respectively, i.e. OUT-type and IN-type. Results are reported both for the individual control of the exoskeleton ankle plantar-dorsiflexion DOF and for that of the exoskeleton knee flexion-extension DOF. EMGs are relative to muscles including: biceps femoris (BF), rectus femoris (RF), semimembranosus (S), vastus lateralis (VL) and vastus medialis (VM), soleus (So), gastrocnemius medialis (Ga) and tibias anterior (TA), i.e. Table 2. Average ± standard deviation of EMG linear envelopes are reported at the bottom of the graph.

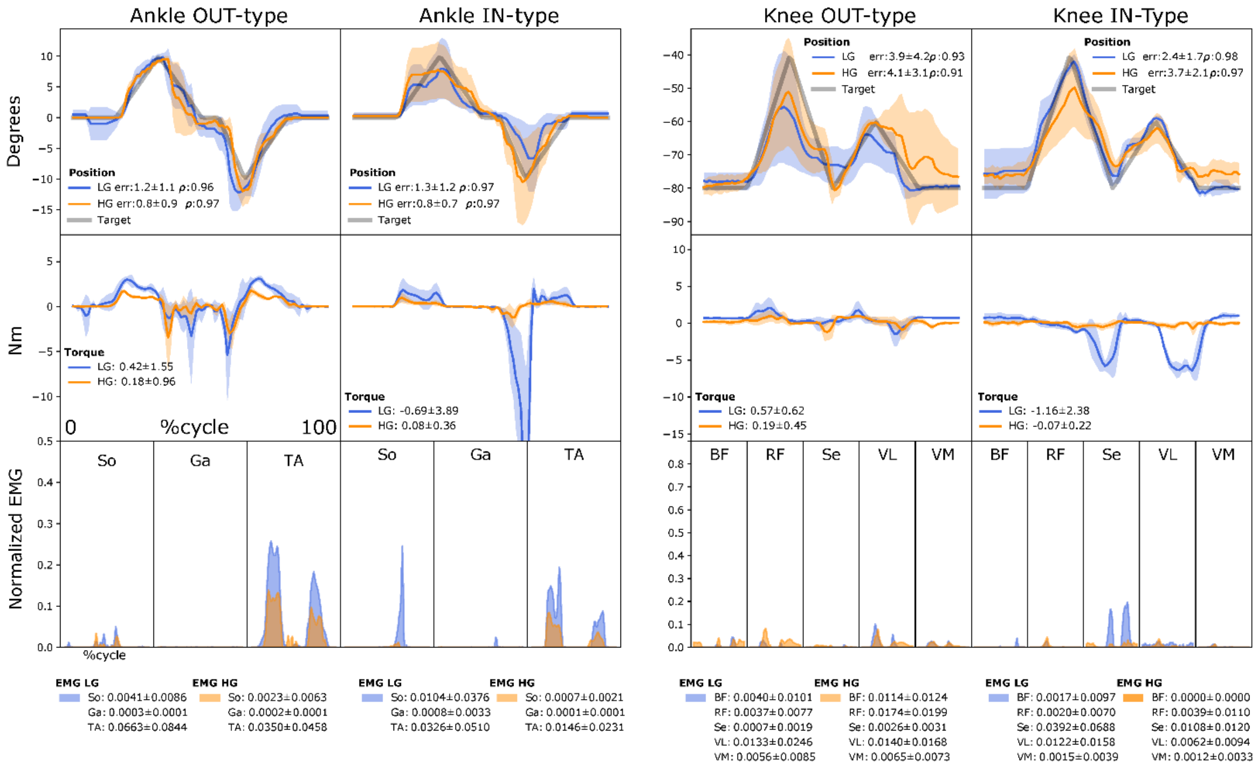

Supplementary figure S2. Tracking task performance during single-DOF tests for healthy subject 4. Exoskeleton joint angular position, electromyography (EMG) data as well as model-based estimates of joint moments (torque) are reported during single degree of freedom (DOF) tasks. Data are reported as averaged across all tracking trials. They are reported for the low-gain (LG) and high-gain (HG) exoskeletons assistance levels and as a function of percent cycle, i.e. where 0% and 100% respectively represents the beginning and the end of the tracking trajectory (target). Results are relative to tests outside and inside of the exoskeleton, i.e. OUT-type and IN-type respectively. Results are reported both for the individual control of the exoskeleton ankle plantar-dorsiflexion DOF and for that of the exoskeleton knee flexion-extension DOF. EMGs are relative to muscles including: biceps femoris (BF), rectus femoris (RF), semimembranosus (S), vastus lateralis (VL) and vastus medialis (VM), soleus (So), gastrocnemius medialis (Ga) and tibias anterior (TA), i.e. Table 2. Average ± standard deviation of EMG linear envelopes are reported at the bottom of the graph.

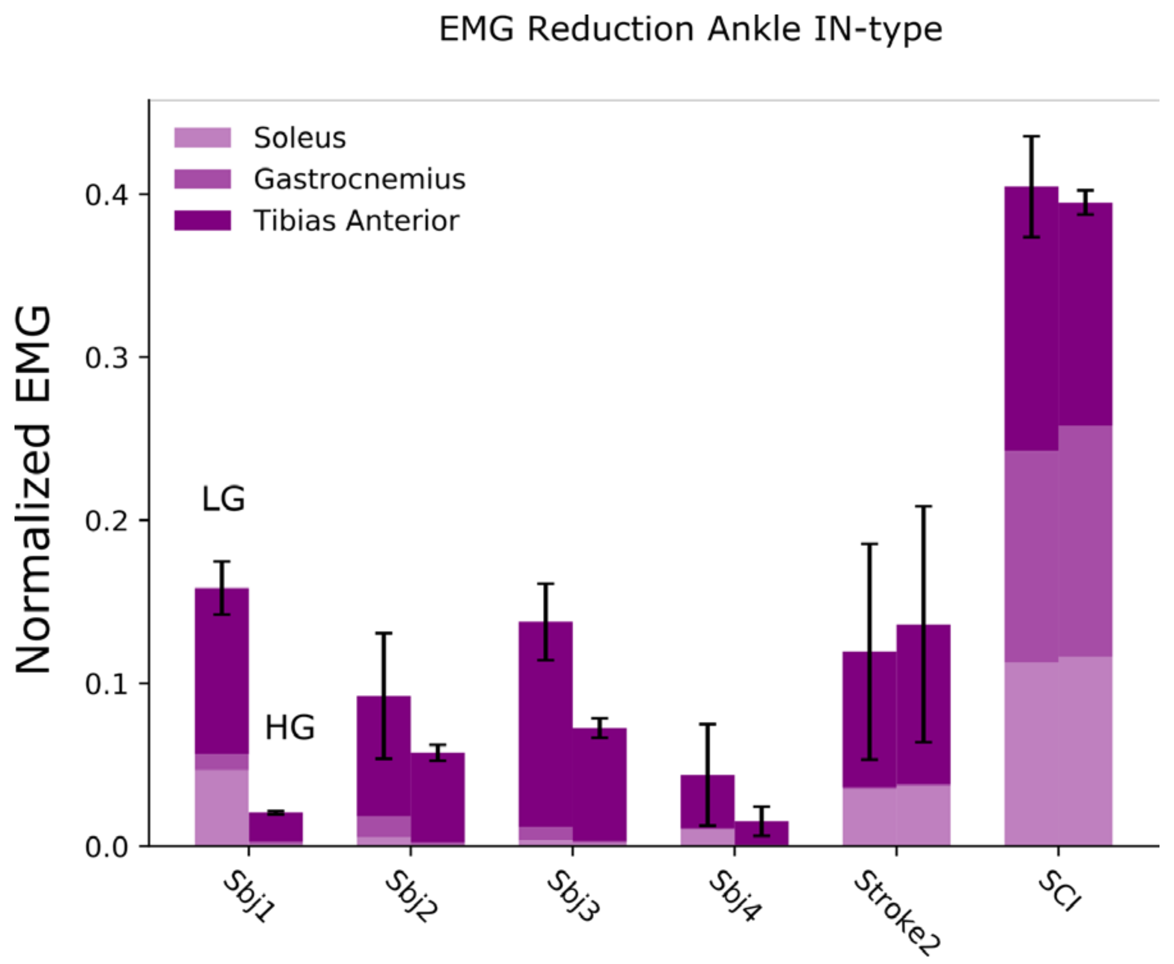

Supplementary figure S3. EMG amplitude modulation between exoskeleton low- and high-assistance levels during single ankle plantar-dorsi flexion, IN-type experiments. Electromyography (EMG) amplitude is consistently reduced when transitioning from low-gain (LG, left-hand vertical bar) to high-gain (HG, right-hand vertical bar) exoskeleton support levels. Experiments were performed while wearing the robotic exoskeleton, i.e. IN-type tests. For each subject (Healthy 1-4) as well as for stroke patient 2 (Stroke) and the spinal cord injury (SCI) patient (Table 1) the vertical bars report mean normalised EMG amplitude stacked vertically for each muscles along with standard devation (i.e. see black vertical lines).

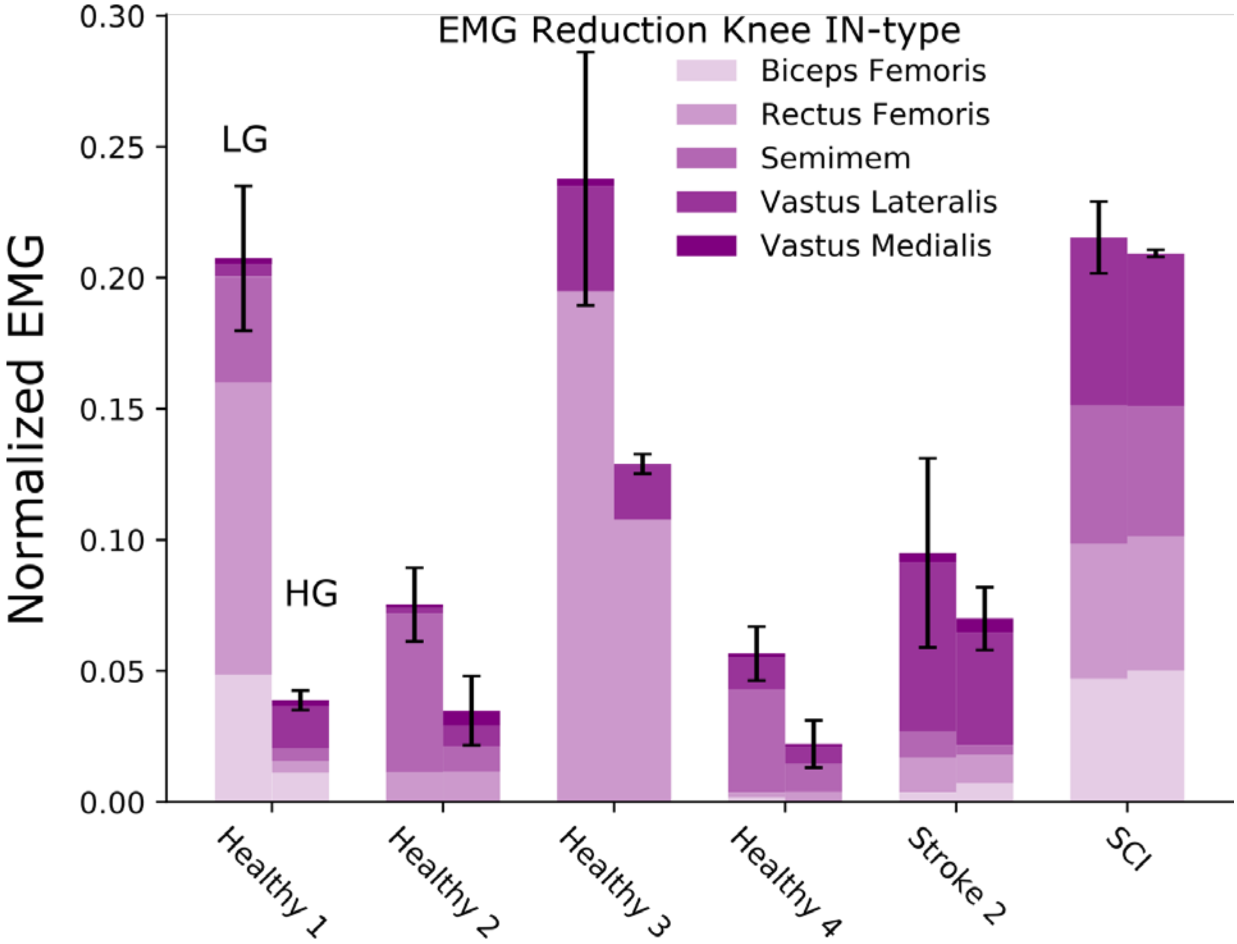

Supplementary figure S4. EMG amplitude modulation between exoskeleton low- and high-assistance levels during single knee flexion-extension, IN-type experiments. Electromyography (EMG) amplitude is consistently reduced when transitioning from low-gain (LG, left-hand vertical bar) to high-gain (HG, right-hand vertical bar) exoskeleton support levels. Experiments were performed while wearing the robotic exoskeleton, i.e. IN-type tests. For each subject (Healthy 1-4) as well as for stroke patient 2 (Stroke) and the spinal cord injury (SCI) patient (Table 1) the vertical bars report mean normalised EMG amplitude stacked vertically for each muscles along with standard devation (i.e. see black vertical lines).

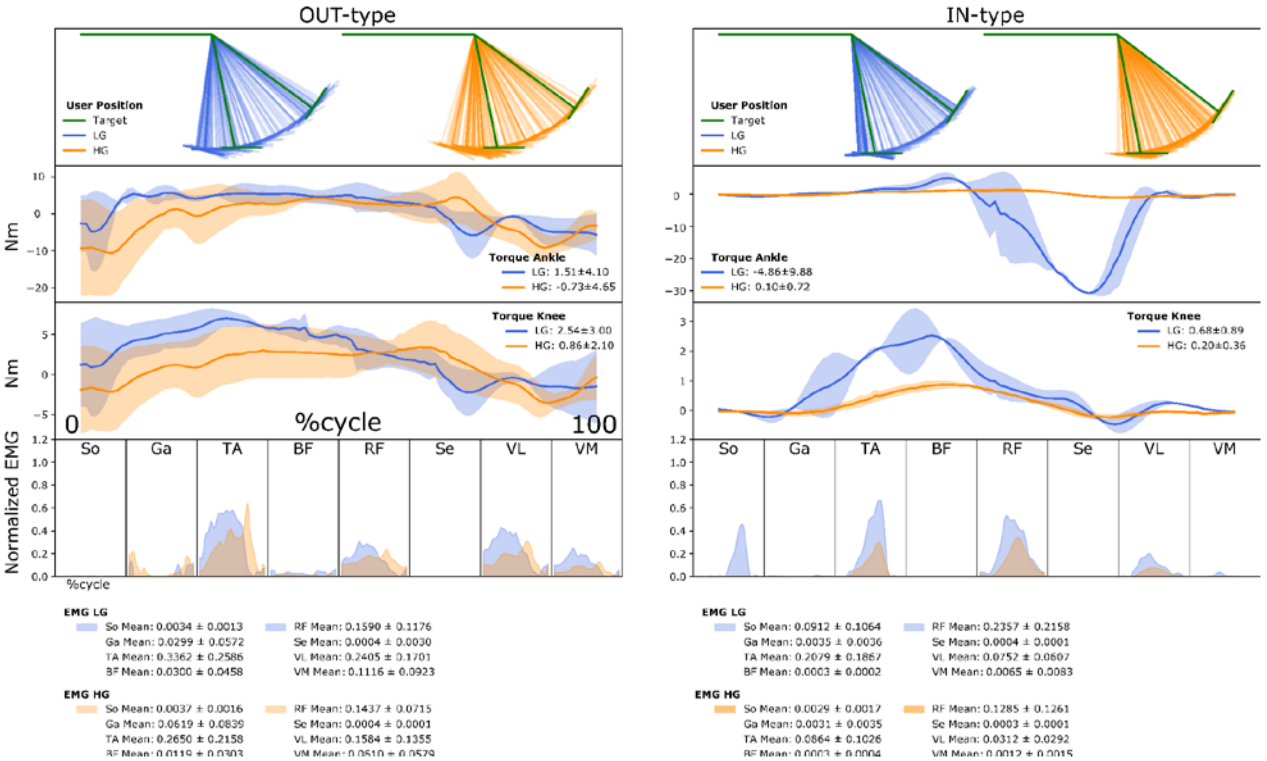

Supplementary figure S5: Tracking task performance during multi-DOF, OUT- and IN-type tests for healthy subject 3. Exoskeleton knee and ankle joint angular positions are reported by means of a stick-figure. The green figure represents the target multi-joint position to be tracked. The blue and orange stick-figures respectively represent the subject's voluntary controlled exoskeleton trajectory obtained using a low-gain (LG) and high-gain (HG) assistance levels. Model-based estimates of joint moments (torque) are reported both about the knee flexion-extension and ankle plantar-dorsi flexion degree of freedom (DOFs). Data are reported as averaged across all tracking trials. They are reported as a function of percent cycle, i.e. where 0% and 100% respectively represents the beginning and the end of the tracking trajectory (target). Recorded electromyography (EMGs) signals are relative to muscles including: biceps femoris (BF), rectus femoris (RF), semimembranosus (S), vastus lateralis (VL) and vastus medialis (VM), soleus (So), gastrocnemius medialis (Ga) and tibias anterior (TA), i.e. Table 2. Average ± standard deviation of EMG linear envelopes are reported at the bottom of the graph.

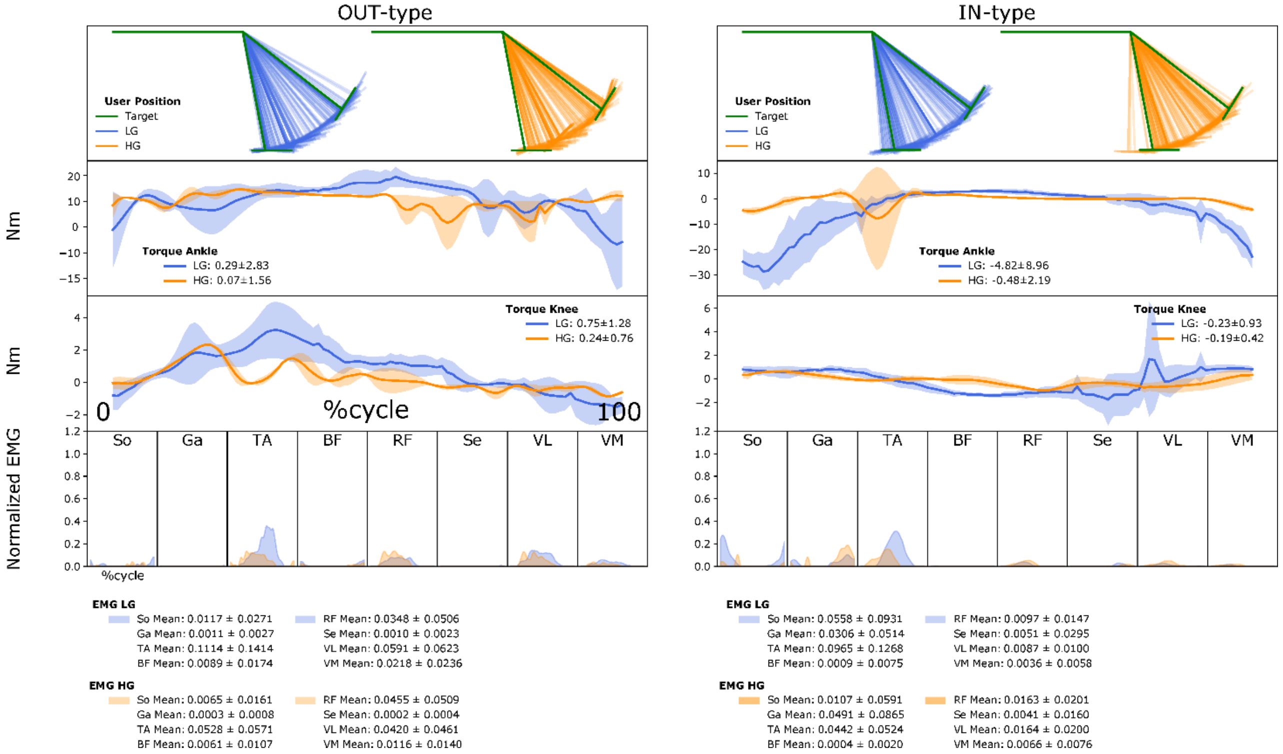

Supplementary figure S6: Tracking task performance during multi-DOF, OUT- and IN-type tests for healthy subject 4. Exoskeleton knee and ankle joint angular positions are reported by means of a stick-figure. The green figure represents the target multi-joint position to be tracked. The blue and orange stick-figures respectively represent the subject's voluntary controlled exoskeleton trajectory obtained using a low-gain (LG) and high-gain (HG) assistance levels. Model-based estimates of joint moments (torque) are reported both about the knee flexion-extension and ankle plantar-dorsi flexion degree of freedom (DOFs). Data are reported as averaged across all tracking trials. They are reported as a function of percent cycle, i.e. where 0% and 100% respectively represents the beginning and the end of the tracking trajectory (target). Recorded electromyography (EMGs) signals are relative to muscles including: biceps femoris (BF), rectus femoris (RF), semimembranosus (S), vastus lateralis (VL) and vastus medialis (VM), soleus (So), gastrocnemius medialis (Ga) and tibias anterior (TA), i.e. Table 2. Average ± standard deviation of EMG linear envelopes are reported at the bottom of the graph.

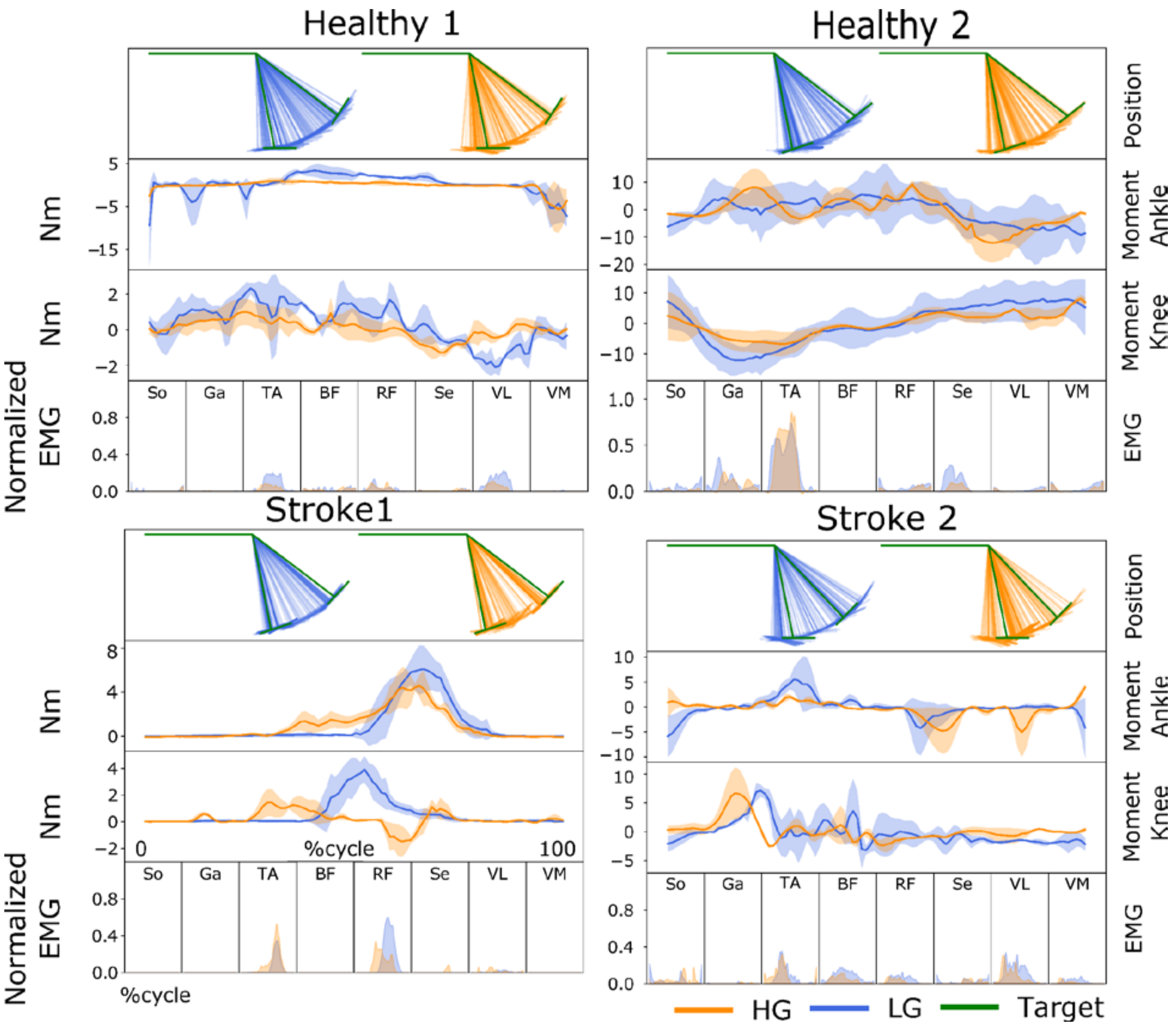

Supplementary figure S7. Tracking task performance during multi-DOF, OUT-type tests. Exoskeleton knee and ankle joint angular positions are reported by means of a stick-figure. The green figure represents the target multi-joint position to be tracked. The blue and orange stick-figures respectively represent the subject's voluntarily controlled exoskeleton trajectory obtained using a low-gain (LG) and high-gain (HG) assistance levels. Model-based estimates of joint moments are reported both about the knee flexion-extension and ankle plantar-dorsi flexion degree of freedom (DOFs). Data are reported as averaged across all tracking trials. They are reported as a function of percent cycle, i.e. where 0% and 100% respectively represents the beginning and the end of the tracking trajectory (Target). Results are relative to tests inside of the exoskeleton, i.e. OUT-type. Data are reported for two representative healthy subjects (Healthy 1-2) and two stroke patients (Stroke 1-2), i.e. Table 1. Recorded electromyography (EMGs) signals are relative to muscles including: biceps femoris (BF), rectus femoris (RF), semimembranosus (S), vastus lateralis (VL) and vastus medialis (VM), soleus (So), gastrocnemius medialis (Ga) and tibialis anterior (TA), i.e. Table 2.

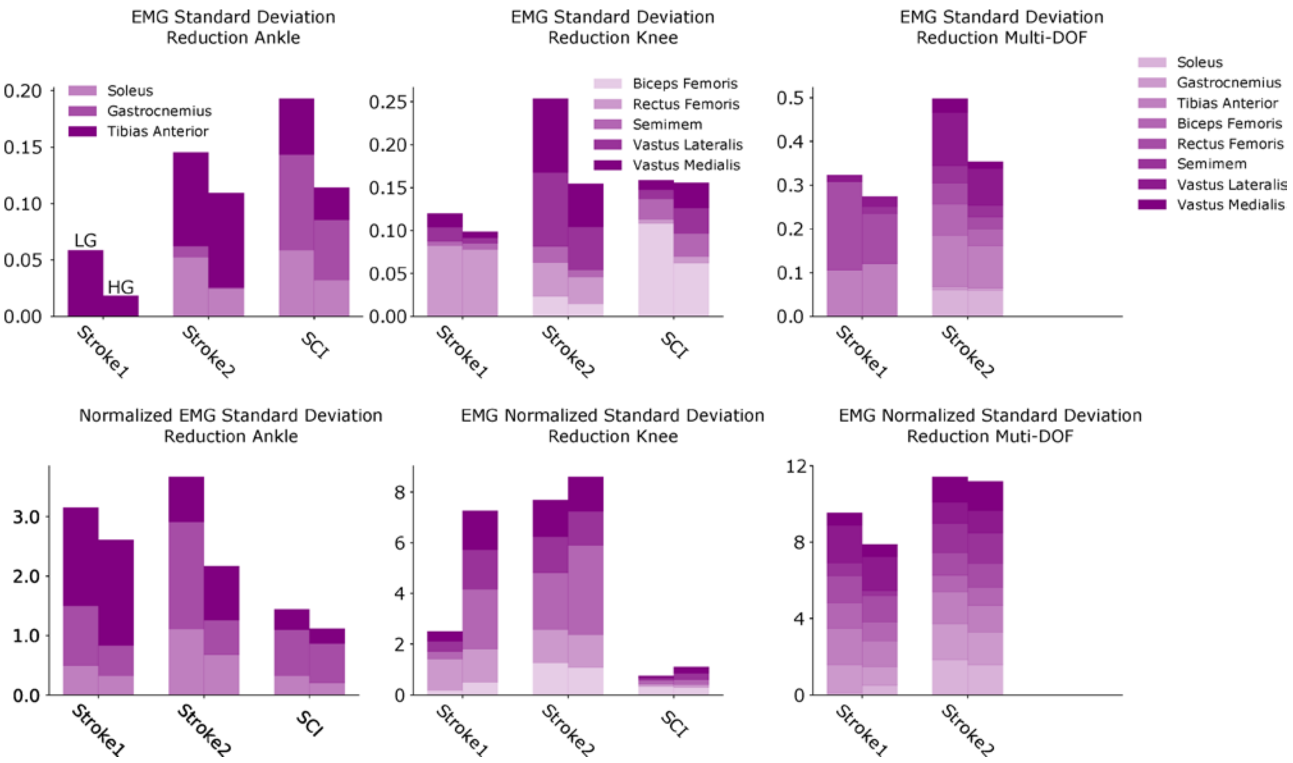

Supplementary figure S8. Standard deviation of the mean EMG amplitude during single-DOF and multi-DOF, OUT-type tests. Histograms report non-normalized standard deviation (top-row) and normalized standard deviation (bottom-row, Eq. 3) extracted from electromyography (EMG) data across of all trials performed during single-ankle control tasks, single-knee control tasks as well as simultaneous ankle-knee control tasks. Histograms are reported relative to low-gain (LG) and high-gain (HG) assistance levels. Data are relative to stroke patient 2 and to the incomplete spinal cord injury patient (SCI), Table 1.

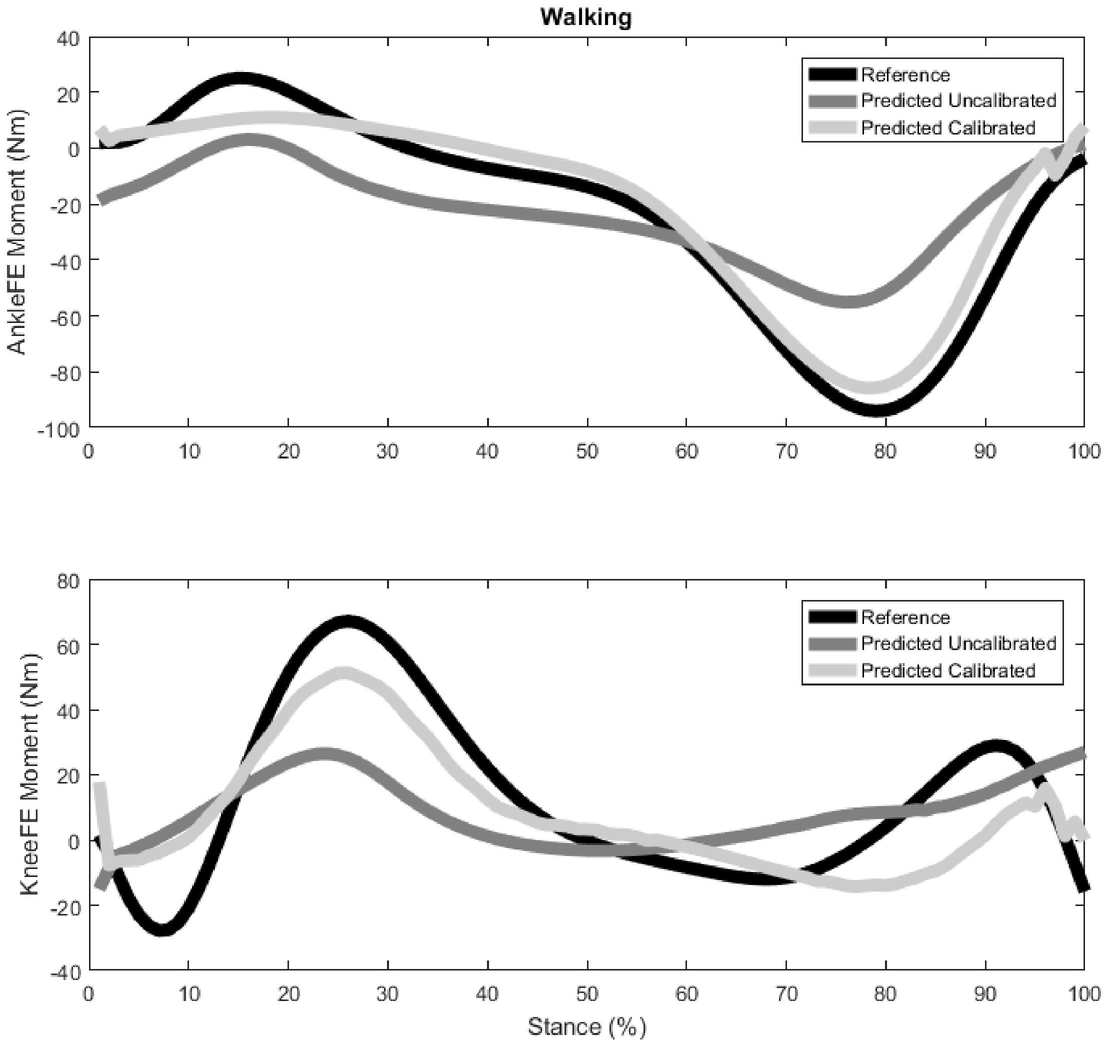

Supplementary figure S9. Predicted moment for the Ankle plantar-dorsiflexion and the Knee flexion-extension using an uncalibrated model. Average of 5 subjects for a locomotion task (fast walking), with in grey line the predicted moment using a uncalibrated model, in light grey the predicted moment using a calibrated model and in black line the experimental moment from inverse dynamics using the OpenSim Software. Data taken from (1).

Sbjs	**Ankle OUT-type N.m (mean± std)**		**Knee OUT-type N.m (mean± std)**		**Multi-DOF OUT-type N.m (mean± std)**				**Ankle IN-type N.m (mean± std)**		**Knee IN-type N.m (mean± std)**		**Multi-DOF IN-type N.m (mean± std)**			
					Knee		Ankle						Knee		Ankle	
	HG	LG	HG	LG	HG	LG	HG	LG	HG	LG	HG	LG	HG	LG	HG	LG
Sbj1	0.81 ± 0.24	1.24 ± 0.87	0.50 ± 0.09	1.31 ± 0.39	0.71 ± 0.11	1.34 ± 0.05	1.42 ± 0.88	2.97 ± 0.20	0.44 ± 0.03	10.6 ± 1.61	0.69 ± 0.03	5.57 ± 0.87	1.69 ± 0.40	6.26 ± 0.33	5.40 ± 4.81	13.7 ± 2.48
Sbj2	1.83 ± 0.24	3.79 ± 0.73	1.22 ± 0.33	3.5 ± 2.1	4.50 ± 0.98	8.33 ± 0.94	6.34 ± 2.09	8.18 ± 1.55	1.25 ± 0.24	3.28 ± 0.79	1.72 ± 0.51	3.97 ± 1.26	5.17 ± 1.72	7.22 ± 1.57	18.3 ± 6.61	14.0 ± 4.95
Sbj3	1.11 ± 0.40	2.18 ± 1.07	1.11 ± 0.56	2.96 ± 0.16	3.72 ± 1.15	4.57 ± 0.38	6.80 ± 2.81	5.55 ± 1.19	0.35 ± 0.05	2.37 ± 1.73	0.20 ± 0.01	0.62 ± 0.10	0.41 ± 0.05	1.19 ± 0.12	0.77 ± 0.09	11.8 ± 0.02
Sbj4	1.18 ± 0.20	2.03 ± 0.38	0.73 ± 0.06	1.03 ± 0.17	0.85 ± 0.03	1.69 ± 0.44	2.17 ± 0.13	3.87 ± 1.29	0.41 ± 0.23	4.01 ± 2.83	0.39 ± 0.03	2.83 ± 0.26	0.57 ± 0.04	1.25 ± 0.54	3.31 ± 3.66	10.9 ± 2.15
Stroke 1	0.82 ± 0.62	2.85 ± 1.67	0.38 ± 0.15	0.69 ± 0.18	0.73 ± 0.14	1.25 ± 0.09	1.72 ± 0.08	2.17 ± 0.46	N/A	N/A	N/A	N/A	N/A	N/A	N/A	N/A
Stroke 2	1.51 ± 1.02	2.98 ± 0.75	0.89 ± 0.44	2.15 ± 0.21	2.10 ± 0.70	2.80 ± 0.34	2.20 ± 0.25	2.50 ± 1.11	1.12 ± 0.57	1.62 ± 0.96	1.31 ± 0.07	2.10 ± 1.00	3.61 ± 0.72	7.21 ± 1.16	3.16 ± 0.98	6.86 ± 0.60
SCI	3.82 ± 1.37	6.65 ± 2.28	3.07 ± 0.77	7.10 ± 0.77	N/A	N/A	N/A	N/A	4.29 ± 0.24	7.14 ± 0.35	1.49 ± 0.08	1.62 ± 0.47	1.15 ± 0.01	1.63 ± 0.00	3.58 ± 0.17	4.88 ± 0.28

Supplementary Table S1: Joint moment modulation across assistance levels. Root mean squared sum of joint moments averaged across all trials for each subject and condition, i.e. see Figs 2-3, 5-6. Data are reported both relative to the low-gain (LG) and high-gain (HG) exoskeleton assistance levels.

	**Ankle OUT-type**		**Knee OUT-type**		**Multi-DOF OUT-type**				**Ankle IN-type**		**Knee IN-type**		**Multi-DOF IN-type**			
					Knee		Ankle						Knee		Ankle	
	HG	LG	HG	LG	HG	LG	HG	LG	HG	LG	HG	LG	HG	LG	HG	LG
**Stroke 1**	5.0	2.0	20	10	5.0	2.5	20	10	N/A	N/A	N/A	N/A	N/A	N/A	N/A	N/A
**Stroke 2**	10	5.0	10	5.0	10	5.0	10	5.0	6.0	3.0	7.0	3.0	6.0	3.0	5.0	3.0
**SCI**	2.0	1.0	2.0	1.0	N/A	N/A	N/A	N/A	2.0	1.0	3.0	6.0	2.0	1.0	6.0	3.0

Supplementary Table S2: Gain used for the assistance for the patients. This gain were used during the experiments were they determined the assistance given by the exoskeleton. This gain were sectioned to give comfortable assistance and were determined experimentally.

1. Sartori M, Maculan M, Pizzolato C, Reggiani M, Farina D, Claudio P, et al. Modeling and simulating the neuromuscular mechanisms regulating ankle and knee joint stiffness during human locomotion. J Neurophysiol. 2015;114(4):2509–27.
